# Supplementary material for: Whole-Body Photobiomodulation Therapy Propels the Fibromyalgia Patient into the Recomposition Phase: A Reflexive Thematic Analysis
Source: Biomedicines. 2024 May 17;12(5):1116. doi: 10.3390/biomedicines12051116 (PMC11117728; doi:10.3390/biomedicines12051116)
Supplement: Supplementary file 1 [file biomedicines-12-01116-s001.zip › biomedicines-3002491-supplementary.pdf]

Supplementary File S1. Data analysis.

The analytical process

Phase 1

Over a 6-month period, Reviewer 1 and Reviewer 3 transcribed a total of 31 semi-structured, audio-recorded interviews for participants A-P (for this stage of the analysis). This consisted of 2 interviews per participant (with the exception of Participant F who was unable to complete mid-treatment interview due to illness) conducted at Treatment Visit 9 and Final Treatment Visit. First visit interview analysis had been completed as part of the previous review.

Reviewer 1 and 3 held a meeting to discuss the review and theme generation process and it was decided at this point that these would be the main reviewers during this phase of analysis. As comments had been made for previous analysis by Reviewer 1 and Reviewer 2 on participants A-E for their First Visit interview and Treatment Visit 9 interview it was decided that for the initial creation of themes for this study, Reviewer 1 and Reviewer 3 would use the analysis of the next 5 participants Treatment Visit 9 and Final Visit interviews to create the themes (excluding participant F until later analysis).

Reviewer 2 was then consulted to discuss the plan for the reviewing phase and agreed it would add depth to review and analyse 5 different patients to those previously used for the theme creation in the previous analysis. Subsequently, once themes were identified it could then be assessed whether review of additional interviews was required to cement the themes.

Reviewer 2 also advised to start with using the overarching themes identified in the analysis of first visit interviews whilst being aware that differences to these may become apparent and they would therefore need to be amended as appropriate.

Reviewers 1 and 3 reviewed and began making comments on the data for these interviews.

Phase 2

Once comments had been made on all interviews by both Reviewer 1 and Reviewer 3, blinded to one another, Reviewer 3 created an individual table per patient per interview to collate the comments of both reviewers and create a summary column to begin categorising before bringing these together to begin identifying initial themes for patients G-K.

It was noted that there were fairly strong patterns coming across all patients and the same themes seemed to be occurring across the interviews for Treatment Visit 9 and Final Treatment Visit just with a larger effect at the Final Visit. The review tables previously created by Reviewer 1 and 2 on patients A-E were then reviewed by Reviewer 3 and demonstrated a further cementing of these themes.

Another meeting was then held between Reviewers 1, 2 and 3 to discuss the themes and subthemes that had been identified. Reviewer 2 suggested that we follow the same process as we previously used for reviewing the initial interviews and that it may be best to try and stick to the overarching themes from the ICF and match those coming out of the interviews where possible to the themes from the first interviews in the previous analysis. They also identified the need to initially capture the changes seen as treatment went on and then look at the mechanisms for this. However, Reviewer 1 pointed out the difficulty now that the reviewers have a better understanding of the processes involved and have experience from the baseline interview analysis that we may be jumping ahead to the processes before selecting themes as the processes are coming through earlier and clearer. Reviewer 3 identified that at times there may be less focus on themes and content of each theme because patients themselves brought processes to the interview and therefore more emphasis may be put on processes. There is a lot of behavioural change interlinked with all themes and we need to feed these themes back into the recomposition diagram. It has been recognised that patients are often hinting within their interviews at recognising it in themselves.

Phase 3

Reviewer 3 created a new table to depict the themes and sub-themes that had been identified and agreed on at the meeting and Reviewer 1 then reviewed and amended the table as required. Quotes from all 16 patients were then collated by Reviewer 1 and 3 and added into one large table to support each of the themes and subthemes with minor changes made to the sub-themes throughout the process to ensure they more accurately represent the data coming through.

Phase 4

Table then sent to reviewer 2 for review and they suggested a new table of analysis to reduce the data and attempt to partly quantify the results. Reviewer 1 and 3 met to discuss and agreed a lot of additional time would be required to populate the new table and were unsure of how valuable quantifying the results would be due to the small timelapse between the treatment visit 9 interview and final visit interview.

Reviewers 1, 2 and 3 then met to discuss suggestions and next steps to move forward. On listening to the opinions of reviewers 1 and 3, reviewer 2 is happy to go with a more thematic approach like the previous analysis of the first interviews rather than translating to numeric data particularly due to the short time frame in which the interviews occurred making the numbers less important and maybe it is more important to take note of the strength and consistency of the themes. It was highlighted by reviewer 1 and 3 that the patient interviews and quantitative scores from the questionnaire did not always appear to

correlate therefore trying to quantify benefits of treatment based on, for example, how many people can be quoted that pain improved is not necessarily fully accurate.

Demonstrating the value of group discussion and all opinions, view points and voices being heard. It was also discussed that including quotes and talking about the negative cases including giving context where possible was important.

The extensive knowledge and experience of reviewer 2 in qualitative research and its analysis provided an invaluable resource in the planning and analysis process whilst having worked with the participants directly and having regular contact with the data reviewer 1 and 3 were very familiar with the data and context which added a useful viewpoint when conducting the analysis and deciding where the main focus should lie.

Supplementary File S2. Participant quotes according to sub-themes.

Theme	Sub-theme	Quotes	Participants
**Body Structure & Function**	Improvement in pain	**Negative cases:**<br><br>**Visit 9**<br>"I'm still getting the…muscle pain in all the usual places and stuff and.. the other day when I came I was actually I felt a lot worse... I haven't noticed any real difference in that in terms of the muscle pain" "because my muscle soreness and stuff it still limits me" [B]<br>"But I think that's 'cause the, the discs they are degenerating kind of and there's no – it's just a case of get on with it [back pain]" [C]<br>"the pain has been worse on the weekends. An' that heaviness." (lack of treatment over weekend) [E]<br>"noticed standing still really hurts my spine but that's because I've got degenerative disc disease as well as fibromyalgia" [G]<br>"I still get a lot of headaches" [O]<br>"I still get my really bad neck and back pain and in my legs" [P]<br><br>**Visit 18**<br>"My chronic pain, um, is not improved as much as hoped" "it hasn't given me the long-term pain relief that I thought it may do" "it hasn't given me the results as in the pain that I would have hoped for" [A]<br>"I think in terms of like sort of er the actual condition and pain and an' tightness in the muscles and that sorta stuff that, that's exactly how it was I think" [B]<br>"at first, I think it was a bit worse um, headaches and pain up in my neck and there was a lot seemed a bit worse" [F]<br>"touch wood at the minute – I'm OK, other than my back" [I]<br>"the pain itself is still as bad, if anything it's probably worse because I've just been diagnosed with arthritis in the hip" [K]	A, B, C, D, E, F, G, H, I, J, K, L, M, N, O, P

		"but like in terms of pain I'm seeing no significant improvement…maybe there has been subtle changes, but not enough for me to notice them if that makes sense" [L]<br>"I mean my legs are still a bit because I've got this hip problem" [O]<br><br>**Quotes:**<br><br>**Visit 9**<br>"The pain isn't as bad days" "I don't have a day without pain. I have a day where I can tolerate pain more." "I've had days where I've felt... that I can do more. The the pain's there but it's like a it's it's not intense so I've been able to do things that I haven't done in a long time" [A]<br>"I was getting a lot of headaches I think it's reduced that. I don't know if it's coincidence but since I've been using it it seems like the frequency of my headaches has been less" "since I've been um here I haven't had any serious headaches compared to usual. So it's just, it's just been manageable" [B]<br>"it's alleviating the pain in me legs" "The leg pains on an evening now I'm not getting the cramp and er the pulsating..... I'd say round about 85% it's, it's reduced" "the pains have started to alleviate" "actually the pain's not that bad on an evening, it's manageable" [C]<br>"I'm not as tender in certain points. So, normally I'd wear a sports bra um 'cause they're softer on my skin and they're not as tight. Whereas I'd say at the end of last week and this week I've been able to put a real bra on" [D]<br>"I think my pain's been a little less. I'm not complaining about it as much" [E]<br>"I don't hurt as much" "I'm still in a lot of pain but it's, it's took at least 2% off so far er of my pain" "the other aches and pains in my shoulders, knees and my feet – that seems to melt away quite quickly" [G]<br>"the sensitised of my skin has gone down. This week I can say is zero and I don't have" "It's not sensitised because normally with fibro it normally burned and could feel crawl sensation and the trigger points would like be on buzzing.. ongoing pain" "I don't get headaches" [H]<br>"my pain's been OK", "journey to Manchester…not been able to do that for years…I've not been able to do anything 'cause I've just been in pain", "my back's always been bad…fibro pain…yeah if you take my back out the equation then yeah it's a lot better…so taking my back out it would probably be like a 5 out of 10 and normally it would be a 9 out of 10" [I]<br>"pain level, I feel better, it's not as intense…a lot of the pain has gone away from my shoulders and my neck completely…I just think – 'why isn't that hurting me?'", "lower back pain…better, better yeah", "I did have a lot of pain come over the last 12 months that's been really hurting me and it's been waking me up in a morning. That hasn't been going on as much…it's definitely helped with my pain" [J]<br>"I mean I've had pain, but nothing like it was" [K]	

		“on one of the days that I come in and there was no pain in my left elbow and I don’t know if that’s ‘cause of the treatment I had 2 days before”, “you know my pain, I went in there [device] and I thought ‘bloody hell, this is good’” [L]<br>“initially I said it was about an 8, 9 but it’s settled to a 7 I would say. It seems to be definitely improving” [M]<br>“I didn’t have pain in places I ain’t had for ages>..for the last 3 or 4 years put it like that…like I say it really does ruddy play me up terrible to like a scale of 8 or 9 when the pain’s really bad, takes me off my feet. But it was the first time it was like 2/3, put it like that”, “it’s like having a metal bar and hitting it and it vibrates…stems from my spine…I feel like a bell, if you hit it it shakes all over and all over here like me hands, my fingers stick…I’ve gone down a notch” [N]<br>“my legs have felt a bit better than normal” [O]<br>“Not significantly, but a bit. I can feel something. Like it is a little bit better... with my pain it’s just a bit better” [P]<br><br>**Visit 18**<br>“since I’ve been um doing the treatment the frequency of the headaches hasn’t been er the same as they were before the treatment – so that’s definitely improving” [B]<br>“less pain in the legs at night” “back’s slightly better. Um sciatica’s still niggling but that’s just off the discs. Um the leg pain on a night’s easier. Um it doesn’t feel like 2 lead weights so to say. Um during the night pain’s not as bad, I’m still getting like cramp” “it’s [pain] eased but not as much as I’d like as I’d have liked it to, but it has it has reduced significantly” [C]<br>“I’ve had far less bad days in regards to my pain” (maybe associated with trigger point injections) “, I would say pain-wise I would say it’s got better” “I do feel less tender” [D]<br>“A lot less than they were at the start, like much less. I’m not using my stick so much, or relying on that, so that’s been really good for me” “Loads better. Like it’s [back pain] still there but I think I said my pain score’s a 4 for last week which is – I can’t remember the last time my pain was this low. Like everywhere aches but it’s not as – I haven’t been complaining that I can’t drive my car ‘cause I’m in too much pain” [E]<br>“my shoulder don’t hurt as much either” “I’m not aching as much, I’m not cracking as much either” “I’m not you know crying out in pain every 5 seconds” [G]<br>“my sensitivity to my skin, nerves pains are suppressed. I’m not having it” “my skin not crawling, not burning, um not getting piercing trigger pain in the trigger pain spots” “but it [period pain] wasn’t like intense [compared to before treatment]” [H]<br>“Pain…so it’s been a lot less, so before this it was about 8 or 9 out of 10 most days and then for the majority it’s been between 5 and 7 out of 10 pre-covid”, “walks…doing a lot of art…which has been easier with less pain” [I]<br>“I think it was about the 11th one [treatment] that I was really getting some relief…the pain was subsiding…wasn’t as much what it normally is”, “neck pain…a lot better than it was, I would say 70%” [J]<br>“I’ve noticed some of the pressure points, mainly on my upper back are not so sensitive anymore and I’m not getting so much pain around the shoulders and the neck”, “the feet as you know I mean these are like 100% better”, “I’m not getting so much	

		pain in the feet, I'm not getting so much pain in the upper body um you know like I say I don't feel like I need like I say injections in the back like I say before the treatment I mean I was ready to have them to be honest and I would quite happily have gone to the hospital and let them do the steroid injections for me I think I'd even um I think I'd even contacted them, the secretary…but now I don't feel like there's any need to rush it to be honest, I don't think there's any urgent need to do it", "going to the toilet in the middle of the night…I used to dread it to be honest just to even put your foot flat on the floor and bear weight on them. It was just like excruciating. But like I say, since this [light therapy] I can't believe there's just no pain there you know and I just don't know what it's done, I mean I just don't know what" [M]<br>"me arms and me hands…haven't been aching so much", "[widespread pain] I do feel better yeah" [N]<br>"I feel a lot better. Very much so. Especially up my neck and my arms and my shoulders…it's [fibro pain] much better" [O]<br>"A lot better, a lot better [pain]" [P]	
	Unanticipated effects	**Negative cases:**<br><br>**Visit 9**<br><br>**Visit 18**<br><br>**Quotes:**<br><br>**Visit 9**<br>"I lose 4 weight. 4 kg" [H]<br>"well I've had effects but they've been like other effects which have been good in a way. Like my skin seems to have improved – honest to God, it's crazy!...like I'd wash my face like you know I'd get all the dry skin off – I've got no dry skin. It's crazy, it's strange…and I had like dark spots which have kinda faded as well…I'd say a few years" [L]<br>"My plantar fasciitis as well I mean it's detailed in my doctors records how much I've suffered with it over the past God knows how many years…years and years and years…and that seems to have just magically, first noticed it end of last week that it wasn't so painful, I could walk about then by Wednesday it's almost gone, it's almost non-existent…I've had injections in both feet, I think I've had 3 steroid injections in the left foot, I've suffered with it quite bed…I started noticing it last week, I was able to get out of bed 'cause that's normally the first thing I feel when I get out of bed…when I first put pressure on my feet, the pain's horrendous…90% better", "I used to have really really prominent lines and soreness in these areas…all my life to be honest…it's started to dry out [since the trial]…the stuff what's at the back of my legs has almost gone…natal cleft seems to be drying out, normally it's like angry and really red and horrible, but this seems to be drying It out for some reason" [M]	H, L, M, O

		**Visit 18**<br><br>"I've already explained the plantar fasciitis…no issues…100% gone in both feet that has now…about 3 weeks in…by week 4 it had totally gone you know…years, its something I've suffered with for a long long time, tried lots of different injections and things", "eczema has totally gone from the creases of my arms…I've suffered it from a child…this is the first time that it's ever disappeared", "and I've got an issue with the natal cleft, I mean that used to be really angry and horrible and nasty but I'd say it's about 80% improved…it doesn't seem as sore and angry and itchy as before", "bags under eyes less as well…they used to be really really dark black to the point where you know you'd think somebody has punched me in the eyes but those have got better as well now, I mean they're not so dark and prominent – something's going on and I just can't explain it" [M]<br>"I haven't had it [numb toes] since I've had been on there [treatment bed]" [O]	
	Reduction in stiffness and improved mobility	**Negative cases:**<br><br>**Visit 9**<br>"the other day for example I just felt really really tight" [B]<br>"I'm stiff when I get out of the machine still" [G]<br>"there's just as much stiffness in the back, my shoulder joints as well, my neck quite a bit of stiffness" [M]<br><br>**Visit 18**<br>"I think in terms of like sort of er the actual condition and pain and an' tightness in the muscles and that sorta stuff that, that's exactly how it was I think" [B]<br>"I'm still really stiff" [F]<br><br>**Quotes:**<br><br>**Visit 9**<br>"the stiffness in me lower body in the morning stopped... I get up now an' I'm fine where usually I was stiff" [C]<br>"I've had less stiffness" "the days I'm having it I will feel less stiff and the day after" "I don't feel as though my muscles are as tight an' as tense as they were before the trial" [D]<br>"my muscles aren't so tight" "[G]<br>"It's as if it's [red light therapy] has gone into my muscles and released them because they're very tight, my muscles…I haven't got those niggles anymore…sitting here now I wouldn't think that there was anything wrong with me", "I feel as if it's muscle…they do feel a little bit softer and more pliable. That seems to have like really eased off" [J]	B, C, D, E, F, G, H, J, L, M, N, O

		**Visit 18**<br><br>"less stiffness in a morning" [C]<br>"during the trial especially the later end of the trial, I don't feel as if my muscles have been so stiff" [D]<br>"I'm having less muscle spasms" "my neck would seize up and I wouldn't be able to move it for days and that hasn't happened at all" "but I'm not so stiff in the mornings" [E]<br>"not as achey, not as stiff" "as soon as I'm out I can feel how loose my joints are again" "out of the 100% that I had – it's down to what, 48%" [G]<br>"I'm not stiff" [H]<br>"neck pain…a lot better than it was, I would say 70% which is good for me because that's where I kept a lot of tension and because of the stiffness in my muscles" [J]<br>"my mobility has improved as well" [L]<br>"I mean I have more range of movement in my shoulders than I've ever had., even my neck, I seem to have more range of movement in my neck than I've ever had…I seem to be able to move better", "the range of movement that I've now got in my shoulders, in the arms you know, I'd say it's increased 70-80%. I certainly wouldn't be holding focus mitts for my wife you know for her to aim and punch at so yeah I'm really happy to be fair" [M]<br>"I feel in me arms and in me hands I feel more flexibility" [N]<br>"it's a lot better... I'm a bit stiff of a morning but after about 5 minutes I'm ok" [O]	
	Improved sleep	**Negative cases:**<br><br>**Visit 9**<br>"I'd started to sleep better but not so much I'd say over the past 2/3 weeks not so much um but again that's not down to fibro, that's down to the PTSD night terrors" [D]<br>"It takes me quite a while still – about an hour or so.... I think it takes my body a long time to properly relax. It feels like everything's really tense." [E]<br>"Sleep is really difficult for me, because I can't get to sleep.... I can't quite seem to find a solution with the sleep. And if I do go to sleep I wake up at 2 2 o'clock and then I can't get back to sleep then" [F]<br>"It takes ages to get to sleep. I go to sleep at like 1am and then I wake a lot in the night. So, this week's been bad 'cause of my back. I can't lie on it so I have to keep turning in the night which wakes me up" [I]<br><br>**Visit 18**	A, B, C, D, E, F, G, H, I, J, K, L, M, O, P

		“Not really a difference with sleep, like I really struggle. I’m on sleeping tablets anyway and then when I wake up I can’t get back to sleep so I don’t think there’s been much change in my sleep but I don’t think I was expecting anything to change because it’s so bad” [I]<br><br>**Quotes:**<br><br>**Visit 9**<br>“I feel more relaxed when I sleep... I can get to sleep, I can get to sleep easier as well, I drop off quicker” [A]<br>“I’m actually sleeping better as a result of it as well” “I just noticed.. in the last couple of weeks.. [I] am getting to sleep.. a lot easier like by half 10/11 I’m ready for bed. An’ when I do go to bed I’m asleep within 15/20 minutes which is, is uncommon for me... I’m sleeping through ‘til about 5.30/6 in the morning so it’s been really good... I’m sure that’s *(points to device)* something to do with it because it wasn’t like that before” [B]<br>“sleep I’ve struggled with for quite a long time. So that’s got better but I think that’s because it’s alleviating the pain in me legs” “Sleep’s been better during the night” “I’d go to bed round about 10. Probably fall asleep round about half 11 just trying to comfortable. Um, I’d be back up at 1... just pain, in my legs. I have to get up and walk round and um yeah I’d go back to bed umm it’d wake me up again... and about an hour, an hour and a half later um then it’d, it’d be like that all the way through the night. It’d be an hour back up and sometimes not going back to sleep.... I’m going to bed about 10 now and probably waking up round about 3ish... [falling asleep] round about half 10/quarter to 11... I probably wake up round about 3ish/half 3... 9 times out of 10 now I can fall back to sleep... so I’m not up as many times during the night” “I haven’t felt that [groggy] for a long time, you know when you get up” “I haven’t seen early morning for a long time” [C]<br>“I feel like I’m sleeping better” “I feel like when I’m going to sleep now my body actually wants to go to sleep instead of like chemically having to go to sleep” “yeah so normally I would have to take my medication. Well, I still take my medication, but normally I’d have to wait for my medication to kick in. Whereas I would say the past 2 weeks I’ve felt my medication start to kick in and I’ve been able to go to bed” “between 10 and 11 normally [normally go to bed]. Whereas now I would say by half past 9 I’m actually ready to go to sleep.. Instead of going to bed and just lying there for hours waiting to be that tired that I fall asleep” “I’m able to wake up an’ just...I feel more alert, like everything seems more vibrant and easy” “, I’m still tossing and turning in my sleep but I seem to be having longer times to sleep” [D]<br>“sleeping a lot better” “I’d wake up a couple o’ times. But since this I’ve just been sleeping.... I’ve been sleeping through the night and not waking up” “I’m not feeling as tired. [when waking up] I’m still tired but it isn’t that really heavy.... I feel like I’ve slept” [E]<br>“I’ve noticed that my sleeping as well has been improved.. like since I’ve been using the machine I’ve been able to relax a lot more” “a couple of days ago I had a whole day of sleep... I just slept all the way through the day and usually I don’t do that... that’s not normal for me so I actually thank that machine for it because it’s like my body needed it” “day I felt so much better for	

		it. More alive instead of been like a walking zombie most of the day" "it's improving, but not improving on the sleeping at the right times at the moment" [G]<br>"it make me sleep like a baby" [H]<br>"Um a little bit, but not a lot…then last week and the week before it was about a 5 out of 10. It wasn't bad bad…I'm not waking up as much…feel more refreshed in morning" [I]<br>"I feel as if I'm sleeping better as well…before I'd go to bed about half11/12 and I'd put one pillow underneath my neck and 3 underneath my knees…then I haven't done it this week [since 6th treatment]", "the pain would wake me up – now I just feel as if I'm going to bed, going to sleep and waking up in a morning. Go to bed same time…and I'll now wake up anything between 8 and 9. Before it was broken sleep, I was in and out of sleep" [J]<br>"the last 3 nights – fantastic. 3 nights full sleep, no problem at all….went to bed 11 o'clock Friday, slept right through the night, Saturday I had a lie in I good up about half past 9, Friday I've done the same, last night I went to bed early…and I slept all the way through until half 7 this morning…which is good for me – not waking up with the pain…long time ago, years ago, I've never had 3 nights in a row. The one night I think it was a couple of weeks ago, I was awake all through the night" [K]<br>"my sleep's improved…for me my sleep has honestly – amazing, absolutely amazing…well before like I'd stay awake, or I'd fall asleep too early…now fall asleep easy…after my first session which was the 20 minute session. The sleep has been consistently good, but that was after the first 20 minute session", "my sleep's better as well, 'cause before it was all over the place" [L]<br>"The first 2 weeks my sleep hadn't really improved, but this week I've managed 6 hours….Sunday, Monday, Tuesday, Wednesday I had a good 6 hours sleep which is good for me – 'cause I'm only managing 3 or 4, so there's definitely something going on" [M]<br>"It's helped my sleep. My sleeps, my sleeps um improved so before I'd go to bed and I'd be lying there until 2 o'clock in the morning not being able to sleep. I go to bed now and I can go to sleep" "I'm not lying there until 2 o'clock in the morning thinking what am I going to do, it's just I go to bed and within half an hour I can go to sleep" [P]<br><br>**Visit 18**<br>"I've noticed improvements in um my sleep and er my mood" "Sleep's been better. Like I'm still um I went through um a week where I was having fantastic sleep um but it seems to have returned er to broken sleep again um but it's better quality of sleep than before" "when I'm awake um in a morning I'm not feeling as tired as I was before er whereas I'd wake up previously and get to work and feel like I need to lie down again er but I don't feel that any more um I feel a bit more positive when I wake up" "falling asleep's improved as well at night. That that's been a big improvement. Um I'm falling asleep within about 30 mins whereas it was taking me like an hour and a half or something like that before" [B]<br>"some nights I wake up at 2 or other nights I can sleep right the way through to 5" "yeah it's still improved a lot" [C]<br>"being on this trial I would say I feel slightly more restful going to bed" "I'm not going to bed and having as many muscles spasms or I'm not going to bed and having to feel restless and uncomfortable and tossing and turning whereas before I would" "I can kinda get into bed and be comfortable and then fall asleep instead of having to think – OK, I've gotta make sure I've taken	

		my pain medications so I can sleep for this long, OK, I've gotta make sure I'm in a certain position so I don't make something feel even worse" "physically I can feel my body's able to rest. Whereas before it wasn't" [D]<br>"I'm sleeping a lot better" "The sleep quality I'm getting has been better" "Like I'm not napping in the day – and I used to. And when I do go to bed I sleep – that's the thing, I'm not being as interrupted" "I haven't had a nap since I started doing this" "I'd say now I'm getting over 8 hours which is great 'cause I used to, I wouldn't go to bed 'til **[04:00]** midnight and then I'd be up at like 6 but I'd be up 4 times in the night just from moving with the pain or whatever. So yeah, I'm getting at least 8 hours" "that I know I'm gonna go to sleep rather than just lie there awake in pain" [E]<br>"my sleep is improved" [H]<br>"I just feel more relaxed when I go to bed. I haven't had to put the pillow round my neck or 3 pillows under my legs to bring my legs up to m ake my lower back flat and I've just gone to sleep on my right hand side…I would say about 5 minutes [to drift off] which is good for me because sometimes I just lie there and I'm just thinking sleep won't come because I'm just wracked with pain", "there's only been like the odd time where I've had just too much either coffee or water before I've gone to bed and it's woken me up to go to the loo, and then I'm quite happy to just drift off again…that doesn't happen normally, for years! I'm like lying there thinking 'I can't have any tablets because it'll mean my dosage over 24 hours", "9 hours straight" [J]<br>"I'd say my sleep pattern's a lot better as well" [L]<br>"Sleeps been a lot better. I've been managing to get to sleep quicker than normal" "About half past 10 and for quarter to 11 I'm asleep whereas before I'd lie in bed a couple of hours tossing and turning before I go to sleep and then after a couple of hours I'd be awake again I'd go to the toilet, back in bed and tossing and turning again. I still get up in the night to go to the toilet but I get back in bed and I go right back to sleep which is really good" [O]<br>"one of the main things that affected me was sleep and that's improved a hell of a lot, it really has, and so now I feel like I've been, although I'm still tired that like, I feel like I've had some rest whereas before I was going to sleep, and I'd go to bed and I'd go to sleep 2 and a half 3 hours later, whereas now I can go to bed, settle better and so I sleep longer" "it's very very rare now I wake up before the alarm" [P]	
	Body feels warmer, enjoy feeling of warmth	**Negative cases:**<br><br>**Visit 9**<br><br>**Visit 18**<br><br>**Quotes:**<br><br>**Visit 9**<br>"I've feeling more warm and my body feels more I dunno like recuperated because of this... my hands, they don't feel as bad"	D, E, J, K, M

		"I've been warm all the time, which is brilliant" "I feel warm all day.. until the next day" "My legs are still freezing but the rest of me's still quite warm [the next day]….. I love it, yeah. Everything seems easier when I'm warm" [E]<br>"Well I feel warmer as well, I feel like hot. Not hot flushing or temperature. I've just felt warmer…I just feel more comfortable and warmer…more relaxed, more happy, happier…I just feel a lot more warmer. Whereas with the fibro and the pain it like drains you of everything, fatigue-wise you know – you're sensitive to cold, achey, miserable…I used to dread walking in the winter because the cold used to – if I got cold it just felt like pain" [J]<br>"it's like the warmth going through my body, it's amazing, it's like being in the sun. 'Cause sitting, lying in the car with sun going sleep in the car which I did of a dinner time the heat helps. And lying underneath that the heat starts to go through all my body because I get cold parts on my body where the pain is which is here, here, here, and here and it heats up all that so I'm nice and cosy and warm for the rest of the day" [K]<br>"it's just like a nice warm summer's day on an Ibiza beach…I just think of good times in there to be honest you know 'cause it feels like literally in the sun, just like you're sunbathing…it ain't got the harshness of a sunbed…it's just nice and warm and soothing in there" [M]<br><br>**Visit 18**<br>"my hands have felt.. they've felt less cold" [D]<br>"I don't think there's any urgent need to do it [injections], whether it's the weather I don't know…the weather's been warmer. I honestly don't know, I'd like to this it's this [device] you know…I mean I've always had the back, the spinal injections and the lidocaine injections…whether it's summer or winter or what you know so" [M]	
	Memory/concentration improved	**Negative cases:**<br><br>**Visit 9**<br>"I still forget things. Like I'll ask you what drink you want and forget by the time I've got to the kitchen" [E]<br>"but my memory is still really bad. Like I don't think it's helped my memory at all" [I]<br><br>**Visit 18**<br>"I don't know if there's been much improvement memory-wise um just because I'm in the habit of always making a list. Um an' I can't really say I've tested not making a list" [D]<br>"my memory's still a bit rubbish to be honest, but I blame that on my medication" [G]<br><br>**Quotes:**	A, B, C, D, E, G, H, I, J, L, M

		**Visit 9**<br>“the day’s I’m having these sessions I feel much better. I feel as if I’m more alert” “I do feel a lot more alert and wake an’ less groggy and foggy” [D]<br>“I’ve been reading more an’ not having to go back so much. ‘Cause I’d have to re-read the pages all the time – so that’s been really nice” “I can watch a drama and follow it without having to rewind all the time” [E]<br>“it’s been a little bit better. It’s not improved a lot um ‘cause I have got a memory of a goldfish sometimes” “I’ve read um half a book ... I’m planning to read the other half in the next few days... I’ve been trying to read a bit more since I’ve been using the machine because I’ve found my focus is a lot better and I can actually concentrate” [G]<br>“It’s [focus] much longer spanned” [H]<br>“Concentration’s a bit better ‘cause I’ll sit and make things. I joined an art class so I’ve been sitting and doing art so I’ve been concentrating on that…it’s on zoom but it’s like an hour and a half so I have to be able to sit for that long. Yeah but that’s been going well as well” [I]<br>“I think that I’m remembering things better…I don’t want to talk because I know what I want to say it just won’t come out and sometimes I’m trying to force those words out, but my speech seems a little more fluid” [J]<br>“And like even my memory. Honest to God, it’s strange because before I’d like grab the key in the morning right…or the heating control or the boiler…and I’d look for it, not forget but I wouldn’t remember where it is – if I’d left it in the chest of drawers or whatever but my memory it’s just – I just seen a slight improvement in my memory as well, so it’s strange” [L]<br><br>**Visit 18**<br>“[memory improved] I think it has a little bit but that’s given me more confidence” [A]<br>“I think my concentration levels have improved a little bit as well compared to before” “I think focus and concentrate a bit longer than before” “I’d start watching er a film something like that and I’d switch off after about 5/10 minutes and and now like um I watched a movie the other day all the way through without looking at my phone or or you know going off” “Memory um I think it’s there is an improvement um not 100% sure um but I I feel like I it is improving” [B]<br>“My memory’s getting better. I’m not forgetting things like I was so much” [C]<br>“I’m not having to go back as much to repeat what I’ve just read” “My concentration’s definitely a lot better” “my memory seems better. Like before I’d ask people what drink they wanted, leave the room and forget everything. And now there hasn’t been a lot of that” [E]<br>“but I am starting to remember a bit more than I used to. It it’s I’d say I’d give it 5% more” [G]<br>“I’ve been reading a lot which I, like before if I read I have to go back and read like the same thing because I forgot what I already read. But I was able to just like read the last paragraph and sort of remember it a little bit more” [I]<br>“[cognition App] I felt as though I was quicker than I initially did it before the trial started. I found it a lot easier to be honest” [L]	

		"I've started using like the rubik's cube…I mean that's something I've not played on since I was a kid", "but concentration in the past I mean it was just a like a zero, I'd just flat line on it basically. You know if somebody gave me something to solve I'd probably spend 2 minutes on it and then just say 'no, I can't help'…but like I say there seems to be a lot of staying power more recently" [M]	
	Brain fog cleared	**Negative cases:**<br><br>**Visit 9**<br>"I don't know whether there's an improvement yet as to like my memory and brain fog – I don't really think that's improved either I think that's near enough the same, I don't know whether like I said before whether there's some sort of crossover between fibro and covid…brain fog got worse with covid" [M]<br>**Visit 18**<br>"I do still get fibro fog" [O]<br><br>**Quotes:**<br><br>**Visit 9**<br>"it's better, better than it was.. it's OK the fog is, but it's it's still there" [A]<br>"an' I feel more, like not as cloudy in my head. So, I wasn't so scared of been on my own.... Like less foggy" "I don't feel as foggy" "I just feel a lot clearer" [E]<br>"actually keep my train of thought which is brilliant.... I'd say about 80% of the time I'd do that [before treatment]. Now it's more like 60%. So it's not as bad but it's still there" [G]<br>"I don't have fog, fatigue in the morning as I would usually have" "it's allowing me to like have a clear focus" [H]<br>"Whereas normally I'd find it really hard. I'd just switch off and drift off. But I've been able to focus" [I]<br><br>**Visit 18**<br>"I don't get up in the morning feeling like a bubble. In like a fog" [H]<br>"you see what happens is I think it's a little bit improved when I speak to people…I feel embarrassed because with the fibro I went right down to using like baby words…to have a flowing conversation I've got to think about what I'm going to say first instead of the thoughts just flowing…it's been a bit better, about 5-10% better" [J]<br>"there's been like the past week my wife's been doing a crossword and she's shouted across 'have you got any input on this like?', and I've thought about it, not had the answers right away, but I've let it tick over" [M]	A, E, G, H, I, J, M, O

	Increased energy - less lethargic and fatigued	**Negative cases:**<br><br>**Visit 9**<br>“sometimes it’s the body’s – the brain’s willing – but the body’s just so, so tired” [C]<br>“I’ve been complaining about the tiredness...I think I’ve been more tired” “the tiredness has been worse” (but sees this as nice as feels ready and able to sleep) [E]<br>“The only issue we have at the moment is like my energy level is... It’s just low” “my energy level is zero” [H]<br>“I feel tired for a little bit [after treatment] but that’s because I’ve been relaxed” “I’m like still tired a lot of the time but I’m getting the sleep” “I still struggle with fatigue but that’s, I’ve learnt that that’s just one of my symptoms” [P]<br><br>**Visit 18**<br>“[fatigue, tiredness and lethargy] quite bad on an afternoon. Um but that’s because maybe I’m just you know I haven’t got, I haven’t got to be anywhere so I think it’s a state of mind but as of Monday when I start a new job I think things will change again there because my mind’s more active” [A]<br>“if it [fatigue] comes on like some of the days it comes on at 11 o’clock and it’s I go really tired to a point where I can’t function so some days I’ve had to – not, it’s not frequent – but there are days where I just go ‘that’s it, I’m done’. But I can’t – if I don’t go back home now I’m gonna have to leave my car and get – ‘cause it’s that. It comes in waves, it’s just, and I can’t fight it when it comes” [C]<br>“I have been waking up quite groggy. But before I’d be groggy and stiff – whereas now it’s just groggy fatigue side, not fatigue as in because my body’s hurting so much fatigue” [D]<br>“because of my new medication, quetiapine I have been a bit more tired and a bit more lethargic” [G]<br>“energy wise it’s been like zero flat” [H]<br><br>**Quotes:**<br><br>**Visit 9**<br>“I don’t feel like when I used to work, um and I’d sit there sometimes and I could drop off when I was at my desk. Um, I don’t feel like I do that now like... it’s been reducing anyway... I do think it’s it’s improved” “More refreshed, more energy” [A]<br>“I think I have a lot more energy” [B]<br>“fatigue’s not so bad now – not as bad as it was” [C]<br>“I feel more alert after the 20 minutes. And a lot less, like my muscles feel less lethargic because they’re a lot warmer, like they’ve been stimulated enough” “I don’t feel as lethargic” “I have felt a lot less fatigued” “before I’d feel really heavy and lethargic when I was waking up, whereas now I feel more awake” [D]	A, B, C, D, E, F, G, H, I, J, K, L, M, O, P

		“I got washed and dressed an’ got in the car an’. An’ that’s a change for me – after doing something from the night previous” “I haven’t been napping” “It’s like my body feels very tired, but my brain’s more awake” [E]<br>“I’ve got a bit more energy” “I’m feeling a little bit more energised” “I’m still feeling achey and lethargic in the mornings still. But it’s not as intense.... I’d say about 10% of that is taken off because I’ve been using the machine” [G]<br>“I don’t have fog, fatigue in the morning as I would usually have” “I don’t have fatigue” [H]<br>“my energy…higher than normally are”, “energy levels…that’s been good. On the 18th I scored it a 9 which for me is very high, and it’s been at a 6 or 7 for the last couple of days…prior to trial…probably about 2” [I]<br>“I’ve got a lot more energy…I think that this [machine] has done something to me, I don’t know whether it’s heightened my energy levels” [J]<br>“On the Friday, I done the house from top to bottom, so work that one out!” [K]<br>“I just feel a lot more energetic like, it’s strange” [L]<br>“I don’t seem to have so much fatigue in my legs, they’re not so tired, same with my arms – they’re not so fatigue-y” [M]<br>“I haven’t felt so tired cus I normally nod off of an afternoon but I haven’t been, I’ve been stopping awake so feel a bit better” [O]<br><br>**Visit 18**<br>“[fatigue now compared to pre-treatment] I think’s improved” [A]<br>“Yeah energy that’s improved... yeah it definitely improved in that area and that has improved my day basically. I’m not getting fatigued um as early as I would before er so you know now I think my fatigue’s kicking in about 3 o’clock in the afternoon whereas before it was about 12/1 o’clock I was exhausted and so it’s much better” “the energy has given me the motivation to do to do stuff” [B]<br>“less tired” “I’m not as drained” “tiredness is here and there. When I’m tired usually the aches and pains start um but yeah overall it’s been an improvement to where I was” [C]<br>“I think that [fatigue/energy levels] might have improved a little bit because as I say I was sitting in the garden for an hour yesterday and I couldn’t have done that” [F]<br>“I felt like I had more energy in my system as well” “my body doesn’t feel so tired” “I’ve got that bit more burst of energy” [G]<br>“not so exhausted and tired” [H]<br>“Energy-wise it was going great until covid. So I scored my energy out of 10, so 10 out of 10 was good so it was like a 9, 8, 6s and 7s so yeah it’s been a lot better” [I]<br>“naps in the day…I haven’t been” [J]<br>“I’ve felt more energised” [M]<br>“I think they [energy levels] have gone up a bit yeh. I’m doing a little bit more than I used to” “I don’t wake up so tired in the morning and so fed up like” [O]	

		"I'd say my energy levels have improved" [P]	
	Increased confidence	**Negative cases:**<br><br>**Visit 9**<br><br>**Visit 18**<br><br>**Quotes:**<br><br>**Visit 9**<br>"I'm not so scared. Even when talking 'cause I got to a point where um you forget what ya' saying... but I just feel like .. I can adapt more now... and I look at people and I can remember.. their names" "I've got a job you know and that's boosted ma confidence as well... I'm just a different person" [A]<br>"I've been able to take him out [dog] for a walk a couple of times after I've had the treatment. On my own as well – so that's quite a big deal for me.... it had been months, and I wouldn't go on my own 'cause I was so scared o' falling..... years.[since going out alone] Gosh that's shocking" "I have been more confident" [E]<br>"*last able to walk the dog prior to trial…*months…confidence" [I]<br><br>**Visit 18**<br>"that's [improved memory] given me more confidence obviously to um looking forward to this job" "I think this treatment has give me more of a push because of my the way I feel right now. I do feel more confident. Confident enough to hold a job down" "I'm walking out of here today a much more confident person than when I first came in" [A]<br>"I'm more OK with doing things that normally I'd kinda second guess because of being in pain or being tired um whereas now I'm like well I do feel OK now so let's go and do it and if I struggle afterwards then at least I've done it" "more free to go and do something that I'd want to and be more present in it instead of like I said worrying about being in pain or recovering" [D]<br>"I've been walking the dog which isn't – and before I'd only go with my Dad because I was so scared of falling over. But I've took him out on my own and without a stick" "I feel more confident. Even when I'm walking without my stick. Like that's a massive thing for me.... they gave me 2 sticks when I was 16. I'm 39 now" [E]<br>"the conversation was flowing you know, and it was quite difficult and challenging really and challenging because the spray on the motorway was up 'here', the visibility was down to about 70%" [J]	A, D, E, I, J, M, N, P

		"the past month I've tried starting using stuff like the rubik's cube, I mean that's something I've not played on since I was a kid, so I've been doing something to test the mind". "I'm happy to try things. I mean, I've even downloaded a few games on my phone…mental games, there's one I'm playing on, 'cross logic, I think there's 'wordle' on here as well…which I wouldn't have done before" [M]<br>"my daughter likes her fishing as well and she's mentioned about coming…well there's a discount on…and I've been on about getting a new reel myself even though I've got about 3 or 4 to be honest with you and I thought 'no, just get it, come on, make a move now, come on, try to enjoy yourself'" [N]<br>"I felt confident enough to be there for him [grandson]" [P]	
	Mood lifted/depression improved	**Negative cases:**<br><br>**Visit 9**<br><br>**Visit 18**<br>"My mood is low because due to a family crisis" [H]<br>"I've struggled mentally this week and that's cus of things going on at home" [P]<br><br>**Quotes:**<br><br>**Visit 9**<br>"I've got like a very positive attitude" "When I first started I was extremely low... But I do feel upbeat so um I just, I just feel very positive, umm, and I push to one side the tiredness" "such a boost" "I feel positive. The pain isn't as bad days so am OK. I'm a positive person" "I just feel as though.. I'm not walking through mud anymore. I feel like I can hold my head up and and you know walk on, walk straight. Um, I'm just upbeat, positive – positive mental attitude. I'm not so down.. I don't you know cry at the drop of a hat, I can take things on. Um, so I just think my mood's improved massively" [A]<br>"I'm feeling um more positive" [B]<br>"More positive than negative [mental health]" "I'm just more happier in meself" [C]<br>"my mood because of the pain is better" "I do feel less depressed because I'm in less pain.. being able to put a bra on obviously is lifting my mood" "more recently 'cause I'm not in as much pain I feel less down on myself" [D]<br>"Just more brighter" [E]<br>"I do feel lighter as well" "my mood itself – it's been quite chipper" "Not been as depressed either. Um not being – not having so many flashbacks 'cause of my PTSD" "I've started to stare more than cry so that's like even though it's not an improvement, it's an improvement in one way 'cause I'm not breaking down in tears I'm just staring... for me, it's an improvement. I mean it's not much of an improvement but it's something" "I'm a bit more bubbly than normal" [G]	A, B, C, D, E, F, G, I, J, K, L, M, O, P

		“my mood…higher than they normally are”, “my mood’s been a lot better as well”, “so I don’t feel as like down either with the pain”, “so I’m scoring my mood between a 7 and a 9…yeah the higher numbers are good on this…normally it’s probably about a 4 or a 5” [I]<br>“this week and part of last week [after 6 treatments] I feel lighter mentally, happier” [J]<br>“it’s definitely helped with my depression. I’ve for to admit I’ve been a lot better in myself, a lot brighter…[first noticed mood lifting] when I was starting the 20 minutes one…brilliant, I couldn’t believe it”, “my depression was bad, really bad I was suicidal – but now I don’t even feel like that now…it’s improving [with each treatment] to a state where I don’t even think about taking extra tablets or doing anything stupid” [K]<br>“and I just feel a lot happier…I feel happier to be honest. Whereas before I was I dunno just like on a downer ‘cause of pain and everything. I’m just feeling a lot happier” [L]<br>“my mood’s ain’t been too bad” [M]<br>“My mood ain’t been too bad, I ain’t been so snappy” [O]<br>“It’s helped a bit with the depression as well because I do feel a bit more. I don’t get agitated as much” [P]<br><br>**Visit 18**<br>“my old mood swing has lifted. Um, I don’t feel like I’ve got a cloud above my head at the moment” “[anxiety and depression levels] decreased. I feel a lot better” [A]<br>“I’ve noticed improvements in um my sleep and er my mood” “mood’s a big one as well ‘cause um that affects you know everything I do and my mood’s definitely improved. I’m a lot happier than before” [B]<br>“mood’s better” “Mood’s been good, yeah. It’s ‘cause I can get things done” [C]<br>“I think I just feel brighter” “I felt happier because I was able to wear something that made me feel better” [D]<br>“I think I’ve been brighter. Like I haven’t been as – yeah I suppose as low really” [E]<br>“it gave me hope whereas before I didn’t wanna live” “I think I do feel a bit brighter I do” [F]<br>“my depression’s improved especially with that *(points to machine)* ‘cause I don’t know what it is about light – it lifts me up, it gives you that sort of – it makes you feel happier in that weird way. I mean literally every time I come home I’ve got a smile on my face” [G]<br>“But very early on, I think about treatment 2 or 3, I just felt lighter and happier…more or less straight away after [treatment] 2 or 3…I think it’s improved at the beginning and then it’s just steadied off” [J]<br>“My depression has improved. Before I started I was suicidal, very suicidal. And it’s improved to the stage where I don’t want to commit suicide anymore” [K]<br>“my mood has improved” [L]<br>“a lot of disinterest in things. Like before I hadn’t got much interest in things like if somebody gave me a newspaper to read I’d just like you know wouldn’t sort of bother with it. If somebody gave me a book to read I just wouldn’t bother with it…I’d	

		certainly say the past month I've tried starting using stuff like the rubik's cube, I mean that's something I've not played on since I was a kid, so I've been doing something to test the mind" [M]<br>"I feel every such a much better, my moods better. I ain't so snappy and a lot better" "my moods lifted as well and I feel good when I'm out walking" [O]	
	Anxiety and agitation decreased	**Negative cases:**<br><br>**Visit 9**<br>"Um, I think my anxiety's still been where it normally is" [I]<br><br>**VISIT 18**<br><br>**Quotes:**<br><br>**Visit 9**<br>"Not really. The only anxiety I've had is finding parking *(laughs).* Yeah, it's been really nice." "I'm not so scared I don't think, even though I fell – 'cause I think because my pain's not in my head so much – it's less – I'm not as jumpy about it" [E]<br>"I don't really think my anxiety levels have been that high recently" [G]<br>"I'm on sertraline 100mg…you know when I was taking the tablets before the treatment I was still getting anxiety, I don't know how to measure if it's improved but I feel a lot happier" [L]<br>"I've been a little bit more tolerant…I tend to be a moody driver when I'm in traffic and stuff like that, I've got a lot of impatience, but I've noticed I'd say the past week I haven't been too easily aggravated" [M]<br>"I love me grandkids…and we look after them quite well…it's like I don't want them in the house…nothing's worse when you feel rough and if someone knocks something and you've gotta get up and you're thinking 'now what I've gotta do'…but yeah I have probably felt a little better that way yeah" [N]<br><br>**Visit 18**<br>"[anxiety and depression levels] decreased. I feel a lot better" [A]<br>"I'd have been anxious about driving on the motorway and stuff like that or er have to plan my route and I wasn't too bad about that so that was good" [B]<br>"in the daytime [anxiety] not as bad" "I'm also less anxious about being in pain. Because I know I'm not in as much pain, I'm not having to worry about being in an extraordinarily amount of pain. So that is making me feel better because I feel slightly more normal than what I was feeling before" [D]	A, B, D, E, G, I, J, L, M, N, O

		"Yeah and my anxiety has been a little lower" [I]<br>"when I'm in pain I don't talk about it, they say 'how are you?' and I go 'I'm fine, how are you?' – and I'm like screaming inside…that feeling has got less [feel less agitated at people]" [J]<br>"not so much anxiety" [M]<br>"I love my wife dearly…but you say things and I think 'I shouldn't have said that'…I ain't as snappy" [N]<br>"I don't feel so anxious now, I feel a lot better like. I feel like something's being done for me" [O]	
	Increased motivation	**Negative cases:**<br><br>**Visit 9**<br><br>**Visit 18**<br><br>**Quotes:**<br><br>**Visit 9**<br>"cause my body does feel different. You know with the warmth – so it makes me wanna try" "'cause my pain's less I wanna try things more" [E]<br>"we're gonna plan to do that [walk dog] on a Thursday" [G]<br>"my motivation…are higher than they normally are", "not been having that nap…some days I've been really tired, but it's 'cause my motivation and my energy's a bit higher…like I don't wanna go to sleep 'cause I wanna be able to do stuff while I'm able to" [I]<br>"done the house from top to bottom…I don't do that! I did the windows, I did vacuuming, I stripped the beds…didn't need to stop for rest", "I'm washing, showering every day…washed me hair last night, teeth are being brushed every day – so the hygiene is getting better as well" [K]<br>"like a lot more active if that makes sense", "I'm not feeling as tired…like I just couldn't be bothered to do like certain stuff, like I'll give you an example like go in the garden or just normal stuff – now, I'm just doing it without thinking" [L]<br>"I was seeing someone for my cognitive issues because of long covid and she noticed that I've got a list of disinterest…I've been using my phone a lot more to be honest. Before I wouldn't even touch my phone but I've started playing some mental games now…you know just to see if it could improve my cognitive and memory recall, it's just something I felt like doing", "I felt like actually going swimming" [M]<br>"I said to the wife, 'if I feel like this at the weekend, I'm going to go fishing…but it will be the first time I've gone probably in 3 years…so just brush the stuff down and see how I feel in myself…and if it's only for 2 or 3 hours I'd like to 'cause if I did I've achieved that" [N]	A, B, C, D, E, F, G, H, I, J, K, L, M, N, O, P

		**Visit 18**<br>"I seem to be cooking more homemade meals... I do seem to want to do that more" "it's just you know routine I would say. Getting into bad, bad ways. And now I'm trying to get out of that" "it has given me a lot of motivation" [A]<br>"I'd like to carry on doing that [swimming]. I think since I've done this that's actually set me off to be able to do something like that" "the energy has given me the motivation to do to do stuff" [B]<br>"that I've got like a – what's the word – a kick start rather than a push if you know what I mean. So that's the plan to get everything done over the summer we can um and then look at fostering later on this year, and then going, whether I go back into schools or whether I stay just as I am and just do the voluntary for a bit" [C]<br>"I'd like to go back to work 'cause I'm still young" [D]<br>"Like I've wanted to talk to people and go out an' visit friends an' things that I would avoid a little bit" "it [going to the gym] still scares me but I think I'd try it now. Like feeling like this I would go" [E]<br>"Just get up to have a bath. So again I think because the weathers nice um I've been thinking yanno shall we have a sit in the garden" "I want to" [do more activities/stencilling with grandchildren] "I'm looking around and I'm thinking I need to do something, I want to do something but I don't know what but I just want to do something, such as the plant pots. Sitting there doing that was a big achievement for me and mopping the floor. But I feel now I want to do a little bit more" "I'm trying [to make an effort]" [F]<br>"25% more motivation" "when I wake up I start to clean" [G]<br>"I want to go back to like doing research study to finish off one of the models ... cus I was like I probably just don't bother earlier in around June July... I think um it's over a week ago I said oh I found myself more alert like yearning to go back and study and to finish my coursework" "I found myself was yearning to go start I said alright this week I'm going to be like use it to like sort out the house and then use my 2 weeks and put my head in and knock out my assignment" "I want to try and do work that can work around my health" [H]<br>"Motivation's been good, it's been higher again" [I]<br>"[Cheltenham to see daughter] I said 'come on then [name]' – that's my brother – I said 'I'm ready to go now so we just got up and got on the M5" [J]<br>"I've sat with her you know and just done nothing…I asked him (physio), I says, 'well my wife goes kick boxing once, maybe twice a week…what if I hold the focus mitts for her?'", "I mean one of the grandkids had a rubik's cube and they'd left it in the house, you know they'd left it on the side board…I've been YouTube looking how to solve it and stuff like that" [M]<br>"I think he [grandson] um he makes me want to do things" [O]<br>"I wanted... as soon as we started this I went home and I was telling my husband and I said I was speaking to the doctor an um and I said we was talking about what I used to do before, ... You said to me, there's something to aim for, that was your words and it's. I'm going to do it at the weekend.... I said if this [treatment] goes to plan that's what I'm going to do" [P]	

	Reduced number and intensity of flares	**Negative cases:**<br><br>**Visit 9**<br><br>**Visit 18**<br><br>**Quotes:**<br><br>**Visit 9**<br>"I haven't had as many bad days an' haven't had days where it's completely like out of the question of me doing anything. It hasn't like been such a prominent thought about being in pain" "I've still had days where I've felt awful but they've not been as awful. So a bad day would be say normally would be like a 9. They've only been like a 7" "I'd probably say have 2/3 a week. Um, but they were like really bad days. They were days when I didn't wanna do anything even though I knew I had to I was having to take extra painkillers just to keep it tol – for me to tolerate. And it was always a thought in my head of oh I'm really in pain like I'm really tired, and my body's really hurting. Whereas I'd say in the past 2 weeks I've only had say 1/2 days of really bad pain" [D]<br>"No flare ups either in the last week as well or two" "I'm not having any like, normally I have like either 2 or 3 good day or bad day. I don't have, um I don't have that at the moment" [H]<br><br>**Visit 18**<br>"when I have a bad day I have a less bad day than what I'm used to um and the don't seem to last as long 'cause sometimes they could last for 2-3 days or if it was a major flare up – a couple of weeks where it only lasted a day and then the following day I'm fine" "They're like mini mini-flares. I have days where I just – everything aches an' it feels like just a how – a mini flare seems to be like what you'd feel when a flare was coming on... But it then it doesn't manifest to anything" "I felt like a flare's coming on but it just never gets there, never surfaces. It's weird." "I cannot remember. [when last flare was. Previously been every 2-3 month] It's been longer. It's been longer this time" [C]<br>"I wouldn't say I've had as many bad days. Or the bad days I have had – they haven't been as bad" [D]<br>"I don't have no flare up [since starting trial] ... I normally get flare up back to back" [H]<br>"I still can't walk very far and you know I still have the days where you just think no, body says no. But it's a lot fewer and far between" "it ain't too bad. When I get them I get them. You know and I din't want to say it's a miracle cure cus it's not but they have been a bit more manageable" [P]	C, D, H, P

	Reduction in time needed to mobilise	**Negative cases:**<br><br>**Visit 9**<br><br>**Visit 18**<br><br>**Quotes:**<br><br>**Visit 9**<br>"More or less 20 minutes, I'm up, I'm up and breakfast done whereas usually it was like an hour and a half/2 hours" [C]<br>"usually I'd lie there for like 20 minutes before I'd even try 'cause o' pain, an' my back feels like it would almost be in spasm. But I've been able to wake up and get straight up" "2 hours. Everything was really really slow [before treatment]...this morning it was an hour. Like I was outta bed, in the shower. I'd had my shower about 8 and got dressed and ready to go" [E]<br>"I wake up I can 'right *dog's name* let's take you out to the toilet', let's go and take myself out – straight away. Instead of going 'hang on, bab, I need to wake up, I need to stretch, I need to do do this', I can literally just jump up and do it now" (used to take 10-15 minutes) [G]<br>"before the trial, probably I'd wake up at half 9 and then I was getting out of bed by 11 but now I've been getting up at like – my alarm goes off at half 9 and like by 10, half 10 I'm out of bed, like up. Yeah that's got better like being able to get out of bed" [I]<br>"I'm still in pain [when wake up] but I feel a bit happy – I dunno it's strange…more refreshed [ready to get on with day]" [L]<br>"I can get out of bed and I can go to the toilet no problem at all, whereas before I'd walk as though I was crippled because the pain was just so intense" [M]<br><br>**Visit 18**<br>"About an hour. That's [time to get going in the morning] improved" [B]<br>"I'm actually up now say I can get up at 6 and be within 40 minutes [previously 2 hours] round and it's I'm not as stiff in the morning" [C]<br>"I can shower and get dressed a lot quicker than I could have beforehand" [E]<br>"when I get up in the morning it doesn't take me 10/15 minutes to get out of bed. I can literally jump up and go to the toilet when I need to... before the treatment it would take me at least 5/10 minutes to get up to go to the toilet. And if I'm desperate then sometimes I could have had an accident" (3 or 4 times a month before trial- none since starting) [G]<br>"I went to bed at half past 12 um last night and I put my alarm on for 8 to come up here because I knew I wanted to wash my hair and um instead of just getting up and doing things I have to think right I have to get up at this time because it takes me this long to do this and this long to do that so I have to plain it more. But I got up this morning, when the alarm went off I didn't	B, C, E, G, I, J, L, M, O

		think oh god the alarms go off I want to stay in bed longer, it was like oh I'm awake now. I need to get up and I need to get up because I'm going to the hospital today" [J]<br>"I've got no issues of a morning now, getting out of bed", "the feet, I mean that used to contribute to about 30% of my problems…it was causing massive discomfort you know and it was just something that was uncontrollable even with injections. I could have injections then literally like 2 or 3 months later the issue would be back – so I don't know, but I mean something, something's gone on" [M]<br>"I'd sit for a good couple of hours before I got up and moved about, but now I can I'll get up, have a coffee, I'll put the washing in and yanno have a potter about" [O]	
	Reduction in analgesics or delay in upcoming procedure	**Negative cases:**<br><br>**Visit 9**<br>"I upped my medication last week but that was I was on the go quite a lot last week um but this week I haven't – I've had extra for the night of gabapentin but that's it." [D]<br>"Fentanyl patch…I'm um on 12 um millgrams at the minute but I wanna reduce it like take it off completely but then I'm still in a lot of pain so I don't wanna be in that much pain that I can't do anything again" [I]<br>**Visit 18**<br>"I was taking 1 [100mg gabapentin] 3 times a day um and now I'm taking 2 of the morning, 1 of the afternoon, 2 in the evening" [D]<br>"I'm still taking the same" [E]<br><br>**Quotes:**<br><br>**Visit 9**<br>"the actual pain, as in paracetamol and ibuprofen I have, I have reduced them" [A]<br>"75mg of pregabalin, 2 tramadol – so they, they've gone now [midday dose]. Um on a morning much the same um I've got me 150 but I've come off, I'm only taking one tramadol... and on an evening... I've come down to 75 pregabalin but 2 tramadol" [previously 150] [C]<br>"I'm meant to take 8 co-codamols a day. But I only take 4 now instead of the 8" [G]<br>"before um it even started because I was taking so much pill pill I was I was getting depressed about it and I just suddenly just missing missing until I found myself not and then when I started I realised um when I started the treatment I realised like oh and the pain, I wasn't feeling the pain, the discomfort. Yeah so like at the moment I'm off it" [H]<br>"And that's another thing – I've only taken morphine twice on a night since the start of the trial…normally 3 times a day" [I]	A, B, C, D, E, F, G, H, I, K, L, M, N

		“I only took painkillers for my headache last night – nothing else…so I had nothing else through the night which is really good” [K]<br>“And to come off the tablets, I’d love to start you know. And I have tried to cut back” [N]<br><br>**Visit 18**<br>“I’ve reduced the medications er slightly” [A]<br>“I’m taking less medication as a result of that [reduced headaches]” [B]<br>“I’ve started reducing my full medication and I’ve started dropping off and reducing the tramadol. I miss sometimes I miss my midday pregabalin out and the tramadol. So but I’m still trying to reduce slowly rather than all at once” [C]<br>“I probably dropped one dose of pain medication [codeine] a day” “So instead of taking 8 I still take 6 but it’s 2 less than I was taking before” [F]<br>“I’m not even taking as much co-codamols. Instead of taking the 8 a day I’m taking 2 in a morning and 2 at night” [G]<br>“oramorph reduced….when I have used it, it’s like in the evening or night time rather than in the day, because I hate using it…because it makes fall asleep and like it drains me so I try and avoid it as much as I can – but at the minute like with the trial it was literally just in an evening if I really needed it, so it’s much better” [I]<br>“My medication dosage has gone down because I’ve realised I was taking too many being depressed and in so much pain that I was overdoing so I have lowered it to what it should be now and not overdosing if you know what I mean” [K]<br>“I never took it on a weekend and I didn’t feel like I was in too much pain. Whereas before when I missed it like a month before the trial started, I was in a lot of pain. So I’ve noticed without the medication, using the light therapy, the pain isn’t as much, it’s not significant, whereas before it was” [L]<br>“I was due an appointment for facet joint injections, I don’t feel I need them, you know whether that’s got something to do with this [device] I don’t know, but at this moment in time…I would re-schedule it, or you know just let me go back to my GP and let them deal with it. I mean I would probably say re-schedule it for 6 months or so because I don’t feel I need it” [M]	
	Feeling more like “old self”	**Negative cases:**<br><br>**Visit 9**<br><br>**Visit 18**<br><br>**Quotes:**<br>**Visit 9**<br>“It feels like I’m getting back to me, back to my old self a little bit” [B]	B, D, E, G, I, J, N, P

		“it’s quite uplifting [being able to wear a normal bra rather than sports bra] ‘cause it kinda feels like something more normal” “I’m still frustrated with my body but these little wins are making me feel slightly more normal” “simple things like curling my own hair. Like that would make him [boyfriend] happy because he can see there’s something I used to do is coming back” [D]<br>“I feel more me, if that makes sense” [E]<br>“I just feel like a little bit more myself” “for the past few years I feel like I’ve been losing myself quite a lot because of the pain that I’m in. I’ve been quite reserved. I’ve been like ‘oh, I can’t do that because I know it’s gonna cause me more pain’, ‘I can’t do this ‘cause of this’. You know you kinda like have to put excuses there so you don’t put yourself through the pain. But now I’m like I’ll try it, and if it puts me through pain I’ll know not to do it next time” [G]<br>“back to making bits of art…yeah and I’d not done any for ages but I’ve managed to…it was around Christmas I did the last one, yeah so it’s been a while” [I]<br>“I can laugh like I used to”, “I felt the old me coming back and I thought – I haven’t felt like that for a long time, years” [J]<br><br>**Visit 18**<br>“I feel like I’m getting a little bit back to my previous self” “I’ve been a bit more back to my usual self and I can get stuff done” [B]<br>“it does kind of make me feel a bit more hopeful. If I can maintain how I have been then it does make me feel more hopeful and more normal” [D]<br>“I just feel like a human being again” “I don’t feel like a disabled person as much in that sense” “I’ve been able to become myself a bit more” [G]<br>“I loved reading before and then I sort of stopped because it was annoying that I had to re-read all the same pages again so I stopped – and yeah I’ve carried on with that. And I’ve enjoyed an art group as a student as well on a Wednesday” [I]<br>“I feel like I used to, before I had the fibro, in my head – with that emotion and mood” [J]<br>“I haven’t felt like that for 3 or 4 years, and you’d pay anything just to be like that again”, “[wife said] ‘you’ve been youself’” [N]<br>“but like now I feel like when I used to” “that was me before fibromyalgia [organising quizzes, big social events]…it was just normal for us” [P]	

**Activities & Participation**	More **willing** to engage in activities with others	**Negative cases:**<br><br>**Visit 9**<br><br>**Visit 18**<br>"I do get quite worried when I speak to people. Like having a conversation now and having to plan out, am I gonna make sense" [A]<br><br>**Quotes:**<br><br>**Visit 9**<br>When asked about reaction to daughter coming over unannounced "I don't get that like anxiety or that you know where you just wanna scream or or run and hide in a corner *(laughs).* Um, I'll just take it on board and deal with what's coming my way." [A]<br>"we haven't seen my friend for like, well over a year now, and it was kind of oh let's go for a bite to eat on the evening. And the evenings are where I tend to struggle 'cause it's like the rest of the day I I've and so I'm kind of on an evening now I'm better than I was" [C]<br>"my Mom's friend came round yesterday so. An' normally if I had a treatment [medical infusion/injection] I wouldn't wanna do anything else like a few days before or a few days after" [E]<br>"I've been helping my roommates out when they need it" "I've been quite friendly to everybody that I've spoken to you know" [G]<br>"Like my back was bad this week but I've still been able to do things", "dog walk…like I was still in a little bit of pain like, but it wasn't to the point where I was taking morphine", "feeling a bit better in myself and wanting to do things. Rather than not wanting to do things 'cause I'm in pain or 'cause I'm tired" [I]<br>"I want to do things [chores]" [J]<br>"To be honest we've been going out, but like the majority of the time's it's public transport when we go out – but like we have been going out…so like for me my mental health has improved" [L]<br>"it would be nice for us all to just go out on Sunday and I'd look forward to that you know…it would just be a drive out and find a nice country pub and have something to eat and a chat and we'd just reminisce of the old times" [N]<br><br>**Visit 18**<br>"I've been able to socialise a bit more er so er I went out for somebody's birthday er whereas before the treatment I just wouldn't have bothered.... it's definitely since coming here 'cause I wouldn't have gone otherwise" [B]	A, B, C, E, G, I, J, L, M, N, P

		"3 more times yeah and I've got – I'm going out tonight and then next week Friday we're going out again" [C]<br>"Like I've wanted to talk to people and go out an' visit friends an' things that I would avoid a little bit" [E]<br>"usually you're like half asleep and don't wanna talk to me [brother]. And now you're rattling off and talking so much that –'. I'm on the phone for 2 hours to him and then he's like '*name* I wanna go, I'm tired!'" "I've been able to hold a conversation with people and not zone out on them" [G]<br>"gets tired towards the end of it but it's one of those things where she keeps going a little bit further", "like a crafty chat sort of session so it's an hour long and we had um different topics so it was like nature based and then each week it was animals or trees or flowers or whatever um and then we were just in our own style producing whatever we wanted in that theme and then the following week you show it and do whatever else" [I]<br>"more interactive with grandchildren…there's been several times where I've took them [karate] you know, I've been chatting with the instructors there", "me and my wife have started playing scrabble you know, that's something again that I've not used in years…I'd normally have said no but I mean these past few weeks…it's something my wife had been playing with the grandkids and then obviously it's come across to me and it's something I've started playing with" [M]<br>"we're all sharing an apartment as well so we can just, I'd have never done that before... before I wouldn't share with anybody apart from my kids... I don't want anybody to see me when I'm at my worst... I ain't gonna be at my worst" "and I said to my husband I'm having um everybody from work round to the house, my team. I said I'm going to bring their husbands and we're going to have a quiz night" [P]	
	Starting or recommencing hobbies/enjoyable activities	**Negative cases:**<br><br>**Visit 9**<br><br>**Visit 18**<br><br>**Quotes:**<br><br>**Visit 9**<br>"I've been able to do things that I haven't done in a long time like yoga and walking me dog" "I manage to get out nearly every day" [A]<br>"I've been twice to the swimming pool now which I hadn't done before" [B]<br>"I'm taking the dog out twice a day now, when it was only once a day before... I can walk a bit further and do more without legs hurting" [C]<br>"I've been moving more. 'Cause I've got a little shih tzu . I've been able to take him out for a walk a couple of times after I've had the treatment. On my own as well – so that's quite a big deal for me... it had been months, and I wouldn't go on my own 'cause I	A, B, C, D, E, F, G, I, J, K, L, M, N, O, P

		was so scared o’ falling” “I’ve done it twice this week an’ I plan to do it when I get home” “me and my boyfriend went to the theatre on Tuesday night.. I was just excited and did it. An’ then still got up and ready on the Wednesday an’ – so I wouldn’t have done that before. If I’d have gone out the night before I would have just had a lazy day and done nothing the following day” “I’ve been reading more" “I can watch a drama and follow it without having to rewind all the time” “Just I feel like I’ve been at the theatre a lot lately. I went to a ballet as well” [E]<br>““I’ve read um half a book ... I’m planning to read the other half in the next few days... I’ve been trying to read a bit more since I’ve been using the machine because I’ve found my focus is a lot better and I can actually concentrate” [G]<br>“and I’ve been able to go on a little walk with the dog on a few days as well which I couldn’t do before….*last able to walk the dog…*months – pain and like fatigue and confidence…after 3rd or 4th treatment”, “I’m back to making bits of art…moulds with glitter and things and photos” [I]<br>“I just feel as though I could pick up on the walking again…I think I could do like 10 minutes but I’m trying to get jobs done instead of going out for a walk” [J]<br>“I bought some hanging baskets and put them in the garden and I done the back garden a little bit which was nice because I haven’t really bothered with it because we’ve got all concrete because of the 3 dogs…my mum bought me one of them 3-seater swings and I started to put things round there and make the garden look nice so yeah I suppose that’s turning into a hobby…that’s new” [K]<br>“I’ve been swimming twice this week as well and that seems to have been easier as well. I felt like actually going swimming…for the weight loss I’ve been going once a week but I went twice this week for some bizarre reason” [M]<br>“I took the dog round the park” [N]<br>“I normally sit down of afternoon, I do a bit of knitting or watch the television and I’m gone but I’ve been awake. It’s been good” “I had my grandson Friday and we went up to Sandwell Valley. He’s only 4 like... I managed to walk round the valley with him so that was good” [not been able to do for about 3 years due to pain] [O]<br>“I had to work in the morning and normally when I have to work in the morning like I’ve been here, I’ve gotta go to work and then I go home and I just I wanna just sit there, but we took him [grandson] to Stourport for the afternoon, and that was really nice so I went on like the little cars with him and stuff like that which I wouldn’t have even attempted before” [P]<br><br>**Visit 18**<br>“yeah we walk, walk the dogs .. just usually for about half an hour” [A]<br>“I’d like to carry on doing that [swimming]. I think since I’ve done this that’s actually set me off to be able to do something like that” “I’m going to Turkey you know I’m going on holiday which is a massive thing ‘cause I haven’t been... That’s a huge thing like er I wouldn’t um have done that but um yeah I feel good so” [B]<br>“We’re going to the theatre next week” “we’re on about going out in June when the weather picks up” [C]	

		"It's just simple little things that I've been able to do and enjoy. Like going shopping for the day with my friends – I don't feel as exhausted afterwards so I've been able to enjoy that more because I'm not having to suffer as much afterwards" [D]<br>"I've gone for walks just for fun with my boyfriend" "I've been walking the dog which isn't – and before I'd only go with my Dad because I was so scared of falling over. But I've took him out on my own and without a stick" ""it's helped with like reading and stuff. I'm not having to go back as much to repeat what I've just read... I've been able to just sit and enjoy reading" [E]<br>"I did some stencilling with my grandchildren" (no stencilled for 5 years) [F]<br>"I managed to get everything sorted [shopping trip] in those 3 hours without needing a rest [previously rested every 5 mins]" "I started reading a book" "Play with *name*. My dog. I've been able to play tug of war with her a bit more and usually that kills my arms off straight away" "I've been able to throw the ball for her [dog], you know play tug of war with her. Got more energy for her" "I've been playing with her which more than what I've been able to do the past year to be honest 'cause the past year I've been just too drained too tired too" [G]<br>"I'm always like not well enough to go to mass in the morning and like I miss out on when she's doing her bit, participation so I push, I remember that morning saying alright" [H]<br>"I've been able to drive up to Manchester which normally I'm in a lot of pain by the time we get out because it's been a long drive. But I wasn't in that much pain and we managed to go for a little walk in the field so that was different because usually I'd have to rest and wouldn't be able to go for a walk but then I was able to so that was good", "being able to go on short walks with the dog which I was struggling to do before, then I have been doing a lot of art like making different things" [I]<br>"I did go for a walk but that was the first walk that I'd gone on since I lost the dog about 18 months ago. So Thursday and Saturday I've gone for a walk" [J]<br>"Like walking as well – I couldn't walk long distances – I'm able to do that, and I'm not getting as tired" [L]<br>"my wife…she's carried on with her kick boxing classes and I've been going with her and using the focus mitts for her…about 4 weeks ago", "I'm still going swimming 2 days a week, sometimes I can manage 3" [M]<br>"I bought a brand new reel about 4 weeks ago, and she said that's the first time I've you do anything with your fishing stuff" [N]<br>"I have been trying to do a bit of walking which I couldn't before" "I've been taking him [grandson] out and enjoyed it yeh, we've had good fun together" "I've been doing a bit of my knitting again which I'd lost all interest in" [O]<br>"like the last few weeks I've been shall we take him [grandson] to the park and I've got on the swings you know with him... I'd never do anything like that before" "we am looking forward to going on holiday because like normally when I'm on holiday, I go on holiday and we hire a car and I lie by the pool or I get in the car and he's like you know we'll be able to do other things. We've always wanted to do the horse riding, there's a sunset horse riding trip and I've booked it!" "and I said to my husband I'm having um everybody from work round to the house, my team. I said I'm going to bring their husbands and we're going to have a quiz night and Martin was like are you sure? I was like yeh. Who's gonna do the quiz night? I said me. And he was like ok" "I remember when we first had this conversation ...I said to you.... there's something I really enjoy doing that I ain't been able to do [bake a cake]" ". I would say it's improved because I can do more with *grandson name*. But whereas before... I'd be the nanny	

		that sits in the chair and he comes and sits on my lap when he needs a rest. To now I do get on the floor sometimes and play with him" [P]	
	More able to do everyday chores/tasks/work	**Negative cases:**<br><br>**Visit 9**<br><br>**Visit 18**<br><br>**Quotes:**<br><br>**Visit 9**<br>"Whereas now, luckily I have got er a job to look forward to... hopefully now I can move forward and and you know, things will be ok" [A]<br>"I don't have to like leave work and go home early. I can get stuff done and then last 'til about 4/5 o'clock" (previously was leaving at 2pm because needed to sleep) [B]<br>"I was starting work round about, the days where I had work I was starting round about 10 o'clock. Now, it's gone to round about um half 8/quarter to 9." "I'm going voluntary [teacher] for the time being, just for a day a week to see how I feel about going back into a school environment" "but that wouldn't have happened before [sorting out bedroom to decorate]" [C]<br>"I'd wear one [normal bra] an' it'd be too uncomfortable after a few hours, an' change or obviously if I had to go out for the day I'd wear it all day but I could – the next day, I could feel as if it was too much... so this week I've wore it all week" "I feel like I'm able to do it [general daily tasks/housework] for longer before I have to stop.. I can do more without having to feel in pain" "I can't remember the last time my hands didn't hurt long enough for me to plait my own hair.. last night I was able to plait my hair" [D]<br>"am less likely to rely on people. I'm trying to do things by myself" "I've been helping my Mom do the housework." [E]<br>"It literally feels like I've gone from doing nothing to doing everything that I can" "a normal shopping trip without the treatment will take me at least an hour. Er with the treatment it could take me even half an hour, maybe even less depending" [G]<br>grip…yeah especially my right hand especially when I grip but yeah it's been OK" [H]<br>"I actually took the trampoline down which I'd been thinking about doing for months but dreading it cus I'm thinking to myself if I start taking that down I'm going to have to stop after about half an hour. And I did the whole thing and it took about 2 and a half hours", "because when you wake up….the first thing I do is open the curtains then go to the bathroom but if I go downstairs	A, B, C, D, E, F, G, H, I, J, K, L, M, N, P

		straight away I start doing jobs and I’m thinking ‘this isn’t like me’. It’s like as if I can do things with a lot more ease…gardening” [J]<br>“When I’ve had it [treatment], I’ve gone back to work and I’ve managed to get through the whole day…which is good for me”, “done house from top to bottom…I’ve not been able to do that much, I mean my windows haven’t been done for about 2 years” [K]<br>“I’m more, I can be more active” [P]<br><br>**Visit 18**<br>“I seem to be cooking more homemade meals” “I think this treatment has give me more of a push because of my the way I feel right now. I do feel more confident. Confident enough to hold a job down” [A]<br>“I’ve been able to stay longer at work” [B]<br>“Like I can decorate when I get home, I can do work when I get home. Whereas before I was getting home and that was it then” “I did 2 days [volunteering] last week, I did 2 mornings um and I’m doing, the following week I’m doing 2 days next week” [C]<br>“say the past couple of weeks I’ve been able to do it [hoovering] and not feel as awful afterwards. So I’m not feeling as exhausted or – I’ve not used all my energy for the day doing one task. I’m able to then... do some like the washing or something without having to say I need help” “I’ve plaited it [hair] a few times. Um my hands have felt – I’ve still had bad days with them where my dexterity isn’t the best um but I do feel I’ve um they’ve felt less cold” [D]<br>“Like I’m hardly using it [stick to walk] at all now. Only if I know I’m gonna be doing a lot of walking - which isn’t really like me” “I haven’t used it [motorised bed] this week, I’ve been able to sit myself up” “I haven’t been complaining that I can’t drive my car ‘cause I’m in too much pain” [E]<br>“up a little bit longer, out of bed a little bit longer” (at times could be in bed for 24 hours) “I mopped the kitchen floor myself... I can’t remember the last time I was able to mop” (years) [F]<br>“for at least a day and a half I feel free to do stuff” “I mean usually sitting in a chair used to kill me off. Now I can sit in a chair for a good hour” “I can walk for a better distance than I used to..... without any sort of pain completely” (from across the room before treatment to length of hospital post treatment) “cleaning up my room I’ve been doing that a lot more” [G]<br>“the shower was helping with relief of pain so because I don’t need that relief I noticed I don’t stay in the shower longer” [H]<br>“walks with the dog…just like over the road and back I would say. I’d say maybe half a kilometre…I could just about walk to the car before” [I]<br>“I’m pacing myself more”, “I washed the car yesterday and vacuumed it…before I wouldn’t have done it in such detail” [J]<br>“Well I’ve seen a significant improvement, whereas before I wouldn’t be able to lift shopping bags – I’d be in too much pain. Now, I’m able to do it” [L]<br>“By week 3 I noticed something’s going on here, and then by week 4 I mean I was able to wake up of a morning just you know stand on my feet, walk to the toiler with no pain…like normally when you’re trying to stand up with plantar fasciitis I mean the	

		pain is initially excruciating. You just physically can't do it but I mean I'm getting no issues with that now", "loading the washing machine you know, I can do it, I manage it a couple of times a week…that's an improvement…[previously] no I don't think so, it would mean getting down on the floor you know and picking stuff up…not a chance" [M]<br>"but you know just different things, when I say different things just little things you notice around the house yourself…I'll get down and get me slippers and stuff like that and I'll think 'oh, this ain't too bad today'…well usually I've got me stick so you can just stick them on", "I just find whatever I seem to do with me hands you know like spreading, that it's not aching as much" [N]<br>"Now normally I wouldn't even contemplate or I'd have wheeled my chair with me but I just sat in somebody else's chair [at work] and sat there for a few hours" [P]	
	Feeling proud/sense of achievement	**Negative cases:**<br><br>**Visit 9**<br><br>**Visit 18**<br><br>**Quotes:**<br><br>**Visit 9**<br>"it's just give me a lot of inspiration" (on getting a new job) [A]<br>"I rang my best friend – I was like I've managed to wear a bra for 3 days an' it was like just such a silly achievement" [D]<br>"I was really proud of myself" (for going on walk on own) [E]<br>"and I've been watering the plants as well in the garden which I've not done before – normally *[name]* has to do it 'cause I can't like bend down and lift…carrying watering can…half full", "since the 1st one, I've done 4 dog walks", "after 1st walk…I felt weirdly proud…I was happy that I'd done it" [I]<br>"and I did the whole thing and it took 2 and a half hours" [J]<br>"I'm just over the moon that I managed to do it [cleaned entire house]…felt like I'd achieved something…it was brilliant to be able to do all that…I felt like the house wasn't dirty around me anymore and I felt like I actually achieved something" [K]<br><br>**Visit 18**<br>"such as the plant pots. Sitting there doing that was a big achievement for me and mopping the floor", "I feel proud of what I've done" "I felt really proud of myself, I did. Really really proud of myself um and made me realise I've been missing out on so much of my life" [F]	A, D, E, F, G, I, J, K, M, P

		"I was crying over the phone to her 'cause I was so proud of myself... I was like proud of myself like to actually push past the PTSD a little bit further" (spoke to a paramedic for 10 mins) [G]<br>"first walk….since about 18 months ago…Thursday was 40 minutes, Saturday was 40 minutes" [J]<br>"focus mitts…it's something new that I wouldn't have done before", "loading washing machine…3 or 4 times in the past since Christmas…it's this pain, it's just this all over body pain and I just feel that some of it has helped from the back upwards" [M]<br>"I did the quiz night and everyone seemed to enjoy it and that was nice because like what made it better... My husband was like sat, he was in a team and I just seen him sat like just seeing it now and he was just sat in the corner like that and he was just watching me and he was like... He was really proud" [P]	
**Environment**	Improved family/friend relationships	**Negative cases:**<br><br>**Visit 9**<br><br>**Visit 18**<br><br>**Quotes:**<br><br>**Visit 9**<br>"So, being in less pain it obviously it does affect your sex life, it does affect your intimacy, it does affect the bond you've got with people – because I'm less grumpy because I'm not in as much pain" [D]<br>"kids irritate me and stuff. I think I'm much calmer and like I just take a different approach... Instead of being angry" [H]<br>Then also we drove…to Manchester. And normally in the car, I don't do very well on long journeys. But I was OK enough to just be able to walk the dogs a little bit just like down the field by *[name's Dad's]* house…which I've not been able to do that for years" [I]<br>"I find things funny, you wouldn't survive in our family without a really bad sense of humour…I've felt like I can joke with my son more, or find things funnier on the television", "the cat got the bird on the patio a couple of days ago…this bird was screeching…my son was devastated because it's his cat and he's very sensitive anyway …and I just said something quite humorous…it sorta took the edge off it all and I just thought – I'd have been feeling like him…in the darkest of moments I can find something stupid or silly to say", "he'll have banter with me", "instead of making a joke out of it…because I have felt miserable being in pain all of the time, but I think you hide a lot from your family and friends" [J]<br>"my mood's changed, I think he's [husband] noticed that 'cause I don't wanna kill him anymore…I was just always on a short fuse and I had to try bite me tongue" [K]	D, H, I, J, K, L, M, N, O, P

		“but we have been going out. It’s just like you know whereas before I’d say no like I’ll say yes now for example shopping and stuff, but I’ll go with them [kids]…before I’d just stay in the car or I’d just not go out at all”, “like go in the garden…it’s just like to sit down to watch the kids play” [L]<br><br>**Visit 18**<br>“conversation was flowing…the thing is we’re big talkers in our family and because of the pain I think that what I’ve done over the years is, because I haven’t had the energy to talk is I’ve just listened more and they just think ‘oh she’s interested’ and it was easy for me to fluff it all, fudge it all by not talking. By me going ‘ yeh yeh yeh’ and I’m thinking I don’t know what to say to that…and sometimes if I’ve had, in the past, if I’ve had a big conversation I’m just shattered”, “[son + daughter-in-law] I just think there’s more banter between us you know” [J]<br>“[sexual activity]…yeah, I mean um 4 times so…yeah I mean in 6 weeks – 4 times, I mean that’s great…before that non-existent, literally non-existent. I mean Like I said me and my wife didn’t have a sex life…oh it’s been years, it’s been years and years”, “I’ve been more interactive with the grandchildren, I mean I used to do what I could with them before, but I’ve been more interactive with them…well normally it’s always been my wife who takes them to karate ‘cause they do karate and kickboxing as well…I mean they normally call me Grandad Grumps but in the past you know I ain’t heard that phrase for quite a while, you know so…so I think they have noticed…they’ve been more eager to come to me if you get what I mean…for example yesterday um I took them to karate, kickboxing but before that because we picked them up from school at half past 1 cus that was their end of day I mean I was sat on my sofa with my youngest grandson for you know 4 hours, they didn’t go to kick boxing until half past 6 but we’re sat on the sofa playing the xbox together I mean that’s something we’ve not done since Christmas day you know so it’s just nice you know building that extra bond” [M]<br>“my wife…we have a lot of time together with the children…but like I say I haven’t been so snappy” [N]<br>“He’s [husband] said how I ain’t so snappy with him” “I’ve been taking him [grandson] out and enjoyed it yeh, we’ve had good fun together” [O]<br>“he [husband] was having some bottles of beer and he said to me the next day he said I felt comfortable enough to have a drink [rather than constantly being on high alert and checking she is ok]”, “I felt confident enough to be there for him [grandson]” [P]	

	Family/friends/colleagues etc. Noticing changes both physically and mentally	**Negative cases:**<br><br>**Visit 9**<br><br>**Visit 18**<br><br>**Quotes:**<br><br>**Visit 9**<br>“me daughter has yeah, she says I’m I’m more like I used to be. She says your confidence I says and ya’ laugh more” [A]<br>“My niece has come round.. she says to me you’re always singing in the morning now.” [B]<br>“you can tell you’re coming back, you’ve got more energy so to say um he says your like quite bouncy” [C]<br>“my best friend,... said that I look less hunched an’ tense” “my Mom and my boyfriend have both said I seem more... energetic like they’ve both said oh well I see you’ve been able to do more things. An’ I’ve seemed less not grumpy but... before if I was in pain I’m the sorta person that I wouldn’t lose my patience. But on the really high pain days it’s easy to get snappy. Um an’ I think ‘cause my pain’s less.. I seem less agitated and frustrated.” “He [boyfriend] said I’m less jumpy in my sleep” [D]<br>“My Mom commented on that like the other day that I haven’t been taking as long as I usually would” “My Dad keeps saying how warm I am when he touches me” “My Dad commented on that – that I haven’t been asking for it [tv programme] to be rewound” “My Mum said I seem brighter” “The hairdresser... she was like what are you having done ‘cause you seem different. An’ I was like what do you mean. She was like you just look brighter an’ you seem more awake.” “he’s [boyfriend] noticed a difference – said I’m walking better” [E]<br>“one of my friends did say that he’s noticed my skin looks different” “he said my face looked like it was glowing a bit more. Like my skin complexion is like getting better” “as well my other mate so I’ve had a couple of friends noticed it [being more engaged in conversation]”[G]<br>“my mood’s been a lot better as well, and other people have noticed it. Like my Mum and my sister have said that they can tell that I’m not in as much pain ‘cause I’m like brighter”, “*[carer]* walk…’cause she said on the way back to the house she was feeling it – but previously she would have had to stop” [I]<br>“we have a very good relationship and he’ll go ‘oh, you’re sharp today’ or…’I didn’t see that coming’. And it’s that kind of thing – I’m thinking yeah, he’s realising there’s something”, “my speech seems a little bit more fluid…I’ve noticed it and I think other people have” [J]<br>“[after first full treatment] they said at work ‘you seem a lot brighter’…I even had music on the other day, singing….even in the office. It was actually nice to feel normal”, “my husband did…’you’ve slept, it’s gotta be getting better’. And my moods have changed, I think he’s noticed that” [K]	A, B, C, D, E, F, G, H, I, J, K, M, N, O, P

		“natal cleft…normally bright red and angry and horrible…a dermatologist, she looked at it…and she said it looks better…I mean you can actually see where it used to be – these red marks are roughly where it normally is and you get a crease line there in here and it’s gone. This arm’s completely gone. This arm’s nearly gone” [M]<br>“my wife says I’ve been quieter…well ‘cause you’re stuck in the house…less nit picking and stuff like that. She said the other day ‘you’ve gone quieter, it’s been better’, so that’s a plus” [N]<br>“my husband’s noticed how I’ve not been sleep” [O]<br>“my husband says that my moods a bit better and he’s noticed that I’m sleeping better because I wake him up when I’m awake”<br>“I was like at work the other day I was dancing, as much as I possibly could and they was like oh what’s happened to you?” [P]<br><br>**Visit 18**<br>“people are saying I’m generally a little bit more happy and um smiley and more jokes” “like there’s an ongoing that I won’t turn up [to social events] and so yeah there was a bit of a cheer when I turned up” [B]<br>“my partner seems to think I’ve picked up which is good” “Mother-in-law’s seen a difference, me best friend – she’s seen a difference.... she said ‘you seem a bit more alive that what you did’. She said ‘you just seemed tired before’ She said ‘you just seemed completely worn out constant’ And she said it was just, ‘it was just like you was fighting – every day was a fight’ and she said ‘but now you seem to have picked up’ which is – and she said ‘you just seem a bit more with it’” [C]<br>“my friends especially. They’ve noticed I seem brighter, and probably more present” “my Mum – she has said.. that I do seem a lot brighter” [D]<br>“My parents, my boyfriend, even the hairdresser. And everyone who’s seen me is like ‘ooh you’re looking really well’.... ‘You’re looking really bright’” [E]<br>“They [grandchildren] couldn’t believe it [suggested stencilling]” “She [daughter]says, you feeling alright mom? (laughs)” (in relation to her mopping the floor) “a few people [family] have said that to me [looking brighter]” “you look nice mum” [F]<br>“she [friend] was like ‘you look better than I do’ [after a big shopping trip]... she was shocked about how much energy I had in me” “she [support worker] said ‘wow, you look 10x better, more energy in your system’... she was like ‘you look fantastic’” “my brother has noticed my voice, ‘cause he talks to me over the phone. He’s like ‘you’ve got more energy in your voice’” “‘wow, you sound different..... usually you’re like half asleep and don’t wanna talk to me. And now you’re rattling off and talking so much that –‘. I’m on the phone for 2 hours to him and then he’s like ‘*name* I wanna go, I’m tired!’” [G]<br>“everyone’s been saying oh you look well” [H]<br>“my mum and my sister and *[name]* have noticed I’ve been more happy like cheerful and I’ve wanted to do stuff, and my energy’s been better…I think it was about the 5th session in or the 6th one”, carer: “it’s enabled you to do more for yourself ‘cause you are in less pain”, “that I’ve been sort of more cheerful, that I’m not sort of in on myself, and then my Mum was saying she feels like I’ve gone from being like a victim to a survivor – because I’m not dwelling on the pain so like it’s made a difference that way” [I]	

		"[wife]…she's noticed a difference in me. She's noticed I've been happier, friendlier…she's definitely noticed a mood change", "my sister-in-law mentioned something like about 4/5 weeks ago that I was a bit chirpier, you know a bit happier", "[crossword with wife] probably thought I wouldn't have been interested in the past…I mean it's something she's noticed because she's obviously noticed me messing with the rubik's cube" [M]<br>"my wife's picked as well with my moods", "my wife…she just said you seem better in yourself, you're much better in yourself…she said you look different and you've been yourself" [N]<br>"my husband was amazed he says god I ain't seen you knitting for a long time" [O]<br>"I was going to work and people would say to me you can tell you're managing your pain better" "my manager said you can see you are managing your pain well because you are coming in looking better... Just like brighter" [P]	
	Less reliance on poor habits formed and recognition of these poor habits	**Negative cases:**<br><br>**Visit 9**<br>"still having a nap at half 3, I suppose because it's a routine though" [K]<br>**Visit 18**<br><br>**Quotes:**<br><br>**Visit 9**<br>"I've noticed that I've not wanted it [alcohol] but it's either habit, or it's in front of the television, or its like 9 o'clock and I fancy a drink 'cause you know what it's like – you sit down and when you've done all your jobs it's like I think your body does it because of your environment around you…I think it's 'cause I feel lighter and better I'm thinking I haven't got to have a good night's sleep…I do like spirits and I don't know whether it's when I was working to get me out of the funk of the day you know to sort of try and get it to switch off…3 times per week for whole of adult life", "since trial…it was just something different. Last night I thought 'oh, I could just have one' and then I think 'no, I don't want it'…I know if I have one it shall be having it for the sake of having it. It's just like I didn't want it. My head was saying 'well, usually at this time you have a gin and tonic' and I thought 'I don't want it'" [J]<br><br>**Visit 18**<br>"I seem to be cooking more homemade meals.. I do seem to want to do that more , it's just you know routine I would say [toast or a sandwich for dinner]. Getting into bad, bad ways. And now I'm trying to get out of that" [A]<br>"it was my comfort, chocolate. It is still. It's my comfort. But I haven't [been lying in bed eating chocolate]. Also, my craving for sugar is not so bad. I don't know if that's because I'm reducing it anyway but the craving for sugar is not as bad" [F]	A, F, H, J, K, P

		“I think that [3kg weight loss during first few weeks of trial] kind of motivate me from there so sort of like um reducing my carbs, eating more veg and um I have like a portion of meat, protein and like watching that amount of stuff” [H]<br>“I can sit down and think of something and my stomach will go over like all butterflies and I’m thinking ‘why am I thinking like that?’. It’s my mind, my thought pattern that’s doing it…I just think that I’ve been used to over the years so much pain, continuous pain, and getting worse over the years it’s just made me quite on edge – but my mood’s a lot better”, “I haven’t had anything like sugary things…not craving sugar as much because not as tired…I think it’s ‘cause I’m not in so much pain now…I think people over-eat if they are miserable in some way or another and because I’ve felt lighter and happier I haven’t kind of gone into that area…I do like chocolate and I was eating a lot of it, I can’t remember the last time I had a piece of chocolate – it was probably about 2 weeks ago”[J]<br>“when I get up now like I get up and I’ll have some breakfast, even if it’s only rice krispies. Whereas before I used to be snoozing snoozing snoozing, I’d get up and I’d have to get ready for work and go out. Whereas now I can get up, cus my husband gets up before me because he has to get out earlier but I’ve been getting up before he’s gone now which is an improvement so now he’s come to get used to that so he gets me a cup of tea” [P]	
**Experience of intervention and trial**	Positive experience - look forward to it, enjoy it	**Negative cases:**<br><br>**Visit 9**<br>“but my back, but lying on the machine hurts it as well”, “trial is good other than lying on my back”<br>[I]<br>“feels neutral prior to treatment” [J]<br>**Visit 18**<br>“it’s OK other than trying to get out of it, like I need someone to yank me up”, “it was fine until I had covid…since covid it gets to about 12 minutes in and then I feel like I’m getting worse so I get like really bad fatigue and feel really drained and wobbly” [I]<br>“you did mention that people had got tingling in their feet, and I’d noticed that before before you said it…but it doesn’t hurt or bother me…I can feel it now and I will tonight and I’ll get up tomorrow morning and I don’t think I’ll feel it…so it’s on the day straight after right up until the night…but then tomorrow morning it won’t be there” [J]<br><br>**Quotes:**<br><br>**Visit 9**<br>“even if it doesn’t take away all me pain, um, what it’s done for me so far, I’m I’m quite pleased. So, yes I am itching to go for the next session.” “I just enjoy lying there. Um, and just letting the mind drift away, which it does. Um, very relaxing" [A]<br>“relieved that something’s actually working” [C]	A, B, C, D, E, F, G, H, I, J, K, L, M, N, O, P

		“Weirdly relaxed and weirdly comfortable.. it’s weirdly soothing” “I enjoy how I feel afterwards... it is kinda like a little treat on the NHS” [D]<br>“this has been brilliant *(points to machine)”* “just excited [to come for treatment]”[E]<br>“I wish I could take that machine home with me and use every day *(laughs)*. It’s one of them um but yeah when I use it, it literally feels like somebody’s melted away all the pain” “positive. ‘Cause I’m like yeah I’m gonna get this done. I’m gonna help somebody hopefully” [G]<br>“It’s beyond my expectation because I thought I would like be in pain still but I seen where it can really help” [H]<br>“but today I lie on my side and it wasn’t too bad”, “prior to session…mainly looking forward to it. Once I’m here I like lying in it, ‘cause it’s warm…it’s like a sunbed…no side effects” [I]<br>“after about 10 minutes I just start to relax…I think it’s because it gets just a little bit warmer and that helps you….can switch off…really relaxed…the rest of the day”, “felt different after 1st 6 minute treatment…haven’t got a word for it…no side effects”, “I haven’t felt like that for a long time, years…and I’m wandering if it’s that machine is like helping me with something”, “positive thinking…and I’ve gone with the flow” [J]<br>“I think it’s just lifted my mood completely…I’m surprised it’s lifted my mood, I’m surprised it’s helped my sleep…in a good way”, “I look forward to it…I didn’t wanna come off today, and the warmth…yeah, I do get excited about coming” [K]<br>“I’m just having the best sleep – honestly I dunno *(laughs)* what it is about this (treatment)”, “more relaxed, settling into the treatment”, “to be honest I’m excited because I have actually seen a difference” [L]<br>“Well, it’s really strange to be honest because there seems to be something working”, “good to be honest, I mean I’m happy to be coming…you know there’s no dread coming to it…you know, I’m eager to come…I enjoy doing it you know” [M]<br>“It’s gone quickly actually” [N]<br>“I look forward to having it” [O]<br>“it sends me to sleep sometimes” “It always helps with my day, it always cheers me up a little bit so I think, because it has helped with my moods though when I know I’m going to... but yeh it always makes me feel a bit brighter” [P]<br><br>**Visit 18**<br>“I feel a bit sad. I’ve actually really enjoyed it. I think it’s been good um not only is it um the benefits you get from the thing it’s like you’re switching off from the world for like 20 minutes, and it’s a routine so that, I think that actually helps as well because you don’t do that at home or at work um just that 20 minutes you’re just kind of taking a break from the day and it actually helps you as well so yeah it’s been I’m gonna miss it” [B]<br>“you feel nice all day and the next day” “Everything just feels better and easier – they’re my overriding comments” “It’s definitely helped me” “it’s made such a difference” [E]<br>“it gave me hope whereas before I didn’t wanna live” “it’s give me hope that there could be something that could come from this that can help me have a better quality of life and improve my health and wellbeing” [F]	

		"I hope it comes through for every body else. Because the research you're doing here it's a, it's a lifesaver" "it's worth a try because like all the pain killers in the world haven't done for me as much as light therapy's done for me right now" [G]<br>"It's a bit hard because it's dipped. Other than that it's been absolutely fine. And I like being in the warm so I've not really been using the fan either", "I found it really relaxing, I was pretty much falling asleep in it", "no other side effects…I think it's all been positive" [I]<br>"the first time that I had the treatment of 6 minutes – on the evening I felt different…there's not a word and I was very aware of it as well but I couldn't pinpoint anything…it just felt different…and I still can't describe it, it's something I've never felt before", "going into the light therapy bed – that really lightened my mood and made me feel happier than I would normally", "I've noticed that…it's like my brain [a switch has gone on], something's happened to it, I don't know how that light has done it – it's done something" [J]<br>"the light therapy – it definitely has probably saved my life", "I've looked forward to coming every time, I mean it's so relaxing and like I say it got me through the day at work…the 20 minutes was absolutely amazing, it was like being in the sun and it really helped with my moods…I mean without this [treatment] I wouldn't be here now" [K]<br>"I feel it has been really good, it's just relaxing you know getting onto the light pod and it's really enjoyable" [L]<br>"Like I say, I think this machine has done the majority of the work because I would never have had the range of movement in the upper body", "like I say, there's something going on because I wouldn't have had this change in my sex life you know, I wouldn't have had you know my sister-in-law, who we sort of get on but, had said I'm seeming much chirpier, she wouldn't have said that", "I think it's improved, something's gone on, I mean like I say my state of mind seems to have changed a little bit, my upper body seems to have improved, lot more range of movement in my upper body, my back…like I say, I've come off the injections…the plantar fasciitis, that's the biggest improvement I've seen, I mean if somebody had said you know 'that will clear, that will help your plantar fasciitis' I'd have said 'get away' you know because I've had it so long and I'm having injections and it had got to the point where I was having injections every 3 or 4 months, and it was getting to the point where the doctor was saying we can't keep giving you injections to your feet 'cause it can cause problems with your tendons and stuff like that…it weren't a solution anyway. But this [light therapy] just seems to have – now I'm able to you know get out of bed in the morning, walk to the toilet and have no pain in my feet, I mean I can get up off this chair", "this [light therapy] has given me something to look forward to you know…it's something positive, it's something worth doing and I think that's what's given me the motivation to come, it's great" [M]	

	Fear of treatment ending	**Negative cases:**<br><br>**Visit 9**<br><br>**Visit 18**<br><br>**Quotes:**<br><br>**Visit 9**<br>“part of me doesn’t wanna see too much of a benefit – for it then to have to just stop” “if I do feel a lot better and then this stops I, I dunno, it’s like giving a child a toy and then taking it back off them” [D]<br>“I did ask the team if it’s going to be like if it’s going to be available to us after the research is finished cus it’s for me it works well and you know at least it will reduce me depending on the amount of medication I have to be taking” [H]<br>“What happens at the end of the trial – can we carry on with it?” [I]<br>“It’s definitely lifted my mood, I’m just dreading when it stops…it’s when it stops, that’s what I’m worried about” [K]<br>“So when the 18 [treatments] are up…but if you start to go downhill a bit is there any plan where you can come in and have a boost?...just probably have 6 and then that would keep you going for another 4 or 5 months probably you know, it’s good relief…is there something where they could book you in for like a fortnight where you could just have like a 3 and a 3 and it’ll put you on a level for another 5 or 6 months you know and if that’s the case could I try and come off a few of me drugs, me tablets. You know I try and look positive I really do” [N]<br><br>**Visit 18**<br>“I’m under no illusion now that I haven’t got the bed to look forward to I will be putting them [analgesic doses] back up again” [A]<br>“it’s become like my little routine now um I don’t know how I’m gonna now like er *(laughs)* once this has stopped” [B]<br>“It’s weird it’s gonna be over, I don’t know what to do with my Monday mornings now” [D]<br>“I’ve had a little bit of relief but how long would it last?” [F]<br>“I mean I’m actually gonna cry today because this is the last one – I wanna take it home with me” [G]<br>“I just wonder if I could have access to it so I don’t have to go back on those pills. Um you know that’s the only thing I’ve got in my head at the moment, fearing to go back on the medication which I don’t want to be on medication for that long” [H]<br>“I just don’t wanna come away from this [light therapy] now and then 3 months down the line I’m going downhill, you know what I mean” [N]	A, B, D, F, G, H, I, K, N

	Would recommend to others	**Negative cases:**<br><br>**Visit 9**<br><br>**Visit 18**<br><br>**Quotes:**<br><br>**Visit 9**<br>"I would recommend anybody having a go" "I hope other people have the chance to you know, chance to do what I've done and I hope it helps other people like it's helping me" [A]<br>"I've told everyone it's lovely" [E]<br><br>**Visit 18**<br>"I'd tell anybody to at least give it a try" [A]<br>"Definitely, definitely do it.[the treatment] I've actually recommended my sister 'cause she's got um similar problems so I mentioned it to her" [B]<br>"I would say to definitely try it without a shadow of a doubt" [D]<br>"I'd tell them to do it without hesitation... it's been wonderful... I'd tell everyone to do it if they get the opportunity" [E]<br>"I'd say give it a go because anything's worth a try" "I would tell anybody and everybody to give it a go." [G]<br>"I've spoken to people about it" "I would have like introduced them to it" "I'd recommend to them" [H]<br>"try it, see how it…if you get one positive from it, it'll help you in that area" , "it was just so easy to come in and use the bed…it's accessible, I know what's expected of me…and the bed, it was so easy, you don't get hot and you don't have any danger from it" [J]<br>"I would definitely recommend it…I've had no issues, it's been easy to use, it's been really relaxing…I mean I've come in, I've had the light therapy and it's got me through the day at work" [K]<br>"I'd definitely recommend it to be honest" [L]<br>"I'd say go for it, you know, got nothing to lose, got everything to gain. I mean I think I've gained something from it….the machine itself I mean it's not uncomfortable, it's not unpleasant you know, in fact it's quite nice, there's nothing to fear from it to be honest…I mean if you gave me the chance to do another 18 treatments in there I'd do it, I'd jump at it you know…I'd say to others 'go for it, don't even hesitate'" [M]<br>"I do feel better than I was and I was glad that I've come onto the project and you know trialled it and I haven't got any issues or anything like that", "advise them yeah…I would turn round and say 'well go for it'" [N]	A, B, D, E, G, H, J, K, L, M, N, O, P

		"I was talking to a woman on the internet the other night from America who's got it... and I told her this treatment I was having and I said it's really helpful" [O]<br>"I think it might help people in different ways. So what I would be saying is don't expect to go on there and your memory, your sleep, your pain will all be improved cus it won't but if it helps you in one part it's worth it" [P]	
<u>**Processes**</u> **– interaction between themes (i.e. doesn't fit one theme alone)**	Not just sleep improvement in terms of objectivity but possibly more importantly positive changes in sleep hygiene and habits (motivated to try not to sleep)	**Negative cases:**<br><br>**Visit 9**<br><br>**Visit 18**<br><br>**Quotes:**<br><br>**Visit 9**<br>"I don't know if that's because I'm feeling more alert during the day that my body's like OK this is tired because we actually need to sleep, not tired because we're fatigued" [D]<br>"I'll be going bed about half 11/midnight [previously] but now I've been going to bed like half 9/9 o'clock some days. An' sleeping through until 7 o'clock" "I haven't been napping. Like I have been staying awake so that when I go to bed I am sleeping" [E]<br>"the latest I go to sleep now is about 4 o'clock.[morning] Which is improving 'cause usually it was like 7" [G]<br>"when I'm in pain in the night…when I wake up in pain I have to take it (morphine) to get back to sleep 'cause I can't sleep, so I'll normally take it at like 3am 'cause I can't lie down again…my pain's been OK", "And I've not been having that nap in the afternoon anymore" [I]<br>"I've noticed the changes within me…I haven't wanted to, I seem to have gone off alcohol. Sometimes…about 3 times a week…I'll have a couple of gin and tonics of a night and I'm just thinking no I don't want that. And that's really significant" [J]<br>"I did have a better sleep I think that night cus of exhausting myself [going out for a walk]" [O]<br><br>**Visit 18**<br>"I'm not napping in the day" "I haven't had a nap since I started doing this" [E]<br>"I'm still spending a lot of time in bed, um but I've had to come out of bed more because I've been coming here" [F]<br>"I'm managing to fall asleep about 2 in the morning and I'm getting up about 12" (maybe due to introduction of quetiapine) [G]<br>"I've gone off quicker, and when I go to bed I am tired" [J]	D, E, F, G, I, J, O, P

		"I used to lie there and think it's going to start hurting in a minute, so I won't bother going to sleep because it's going to wake me up.... I've felt like I can relax more [since starting treatment]. Like I'd get in bed and just, just relax whereas before I'd get in bed and you'd be like, and you'd be like mithered" "before the trial I used to go to bed at half 10, 11 and go to sleep about 2 and I'd lie there on my phone or looking, doing whatever and then I'd get up still, I'd get up at quarter to 7 and the alarm would go off and it would be like snooze, snooze, snooze all the time whereas now I go to bed about half 10 quarter to 11 ish and I, and like by quarter past 11 I'm asleep" [P]	
	Benefits of improved pain and sleep having big knock-on positive effect on mood, energy, confidence, motivation and ability to cope (and vice versa)	**Negative cases:**<br><br>**Visit 9**<br><br>**Visit 18**<br><br>**Quotes:**<br><br>**Visit 9**<br>"I'd wake up before feeling like um I'd been beaten up in my sleep and now um I'm having a decent sleep am feeling better and if I do go swimming it's gotta be first thing in the morning I can't go later in the day" "I think because I'm sleeping better um my mood's improved in a morning when I wake up" [B]<br>"I just felt like a bit happier and I wasn't in as much pain so I thought I might as well try while I can and then if it doesn't work it doesn't work but at least I tried", "even though I've not been sleeping, and waking up still I don't feel as down the next day. Normally I don't wana get out of bed because I'm like so fatigued. But because I've felt a bit better like pain-wise I've been in such a better mood so I've been doing things that like I normally wouldn't do" [I]<br>"taking trampoline down…I listened to stories through Sandwell library and I have that on so it sort of distracts me from the pain. And I was listening to it and I was just doing it – before I would have had to have stopped…I think that if I get pain I sort of adjust my body…if like my fingers hurts, I used a different tool or spanner or something that wouldn't hurt me as much" [J]<br>"but for me to get through a whole day of doing what I did [cleaning house] – I mean I think it was about 6 o'clock at night time when I stopped and that was from about half 8 in the morning" [K]<br>"work…to be honest I've been feeling less tired. Full stop" [L]<br>"I actually felt like going swimming…I felt like I had more energy – I went Thursday and Tuesday…felt fine after it" [M]<br>"Everything's going in the right direction" [N]<br>"when it's all bad, it's all bad. When you've got little bits that you can cling onto that ain't so bad then that makes it all not so bad" [P]	B, E, I, J, K, L, M, N, P

		**Visit 18**<br>“when I’m awake um in a morning I’m not feeling as tired as I was before er whereas I’d wake up previously and get to work and feel like I need to lie down again er but I don’t feel that any more um I feel a bit more positive when I wake up” [B]<br>“I’m sleeping a lot better. So I think that’s helping with the pain as well ‘cause I’m waking up feeling like I’ve had some sleep. An’ I’d wake up even if I’d slept all night before and wouldn’t feel at all refreshed. So I think that’s helped with the pain” [E]<br>“so because I’ve not been in as much pain…I’ve been more happy like cheerful and I’ve wanted to do stuff and my energy has been better as well so yeah because I’ve been in less pain it’s been impacting other bits as well” [I]<br>“Saturday – I drove down to Cheltenham and went walking with my daughter…[previously] I know that I would have been in a completely different state of mind, mood, pain, knocked out for a couple of days…[previously] it’s like the build up to it and then being there…but Saturday I just got in the car, I said come on then *[name]* – that’s my brother – I said ‘I’m ready to go now’ so we just got up and got on the M5 and chatting all the way down there…the conversation was flowing you know” [J]<br>“It’s really helped 100% with the depression, OK – not so much with the pain, but at least I know I can get on with life now because it has calmed the depression down” [K]<br>“things have been easier”, “focus mitts…that’s been helping, you know I don’t seem to have as much stiffness in my shoulders…it just seems that the pain in the upper body just seems to have been reduced, it doesn’t seem there anymore, the joints feel easier, the neck feels easier to move”, “[nearly 10,000 steps before] I doubt it, not in a day, not in a day…I was with my wife at kick boxing I believe…so whether it was walking upstairs to he gym through the car park you know I think we’d gone shopping that day as well”, “[rubik’s cube] you know it’s something I’ve never been able to solve. I still ain’t solved it yet, I’ve done the 1 face and the bottom 2 rows but I’m just like scratching my head over it to be honest, you know but before I wouldn’t have even tried”, “I seem to have more staying power with things, I mean if I couldn’t do something I’d just put it down and just ignore it but I seem to have persevered with this rubik’s cube” [M]<br>“say from a scale to 10 when I seen you, 10 I felt you know I’m on a 10, and like now I’m on a 7 [overall condition], that’s the best way to put it”, “I still find it difficult to get around but I still feel a lot better than I do, put it like that” [N]	

	Improvement in memory/concentration helping with ability to do and enjoy hobbies	**Negative cases:**<br><br>**Visit 9**<br><br>**Visit 18**<br><br>**Quotes:**<br><br>**Visit 9**<br>"I've been reading more an' not having to go back so much. 'Cause I'd have to re-read the pages all the time – so that's been really nice" "like I can watch a drama and follow it without having to rewind all the time." [E]<br>"I've read um half a book ... I'm planning to read the other half in the next few days... I've been trying to read a bit more since I've been using the machine because I've found my focus is a lot better and I can actually concentrate" [G]<br>"it's like the pain's at front on mind so there's no creativity getting though so when less pain more herself" [I]<br><br>**Visit 18**<br>"[memory improved] I think it has a little bit but that's given me more confidence" [A]<br>"concentration – yeah, I can sit down and read a little now" [C]<br>"it's helped with like reading and stuff. I'm not having to go back as much to repeat what I've just read... I've been able to just sit and enjoy reading" [E]<br>"I was able to like just read the last paragraph and sort of remember it a little more so then I've just been reading like more than I did before" [I]<br>"[rubki's cube] enjoying trying to solve a problem…before I just couldn't be bothered- books, newspapers…would have just walked past it probably yeah" [M]	A, C, E, G, I, M
	Self-awareness and insight of interlink between symptoms	**Negative cases:**<br><br>**Visit 9**<br><br>**Visit 18**<br><br>**Quotes:**	A, C, D, E, I, J, K, L, M, N

		**Visit 9**<br>“it hasn’t removed all my pain but what it has done is made it more positive [her mindset] and easier to deal with the pain” [A]<br>“More positive than negative [mental health]. Um but I think that’s because the pains have started to alleviate. I’m starting to be more active, so it kind of, being out there... seeing more people as well I think” [C]<br>“I have learnt to do everything in pain but after a little while so if the bad days are like continuous it really affects my mood, my energy levels – because it’s constant and there’s no break. Whereas only having one or two to me that’s a big thing because I can kind of manage having 1 or 2 – and feel less…hated on my own body” [D]<br>“Yeah it has [changed lifestyle]. For the, for the better” [E]<br>“then out of 10, I’ve scored my mood, my sleep, eating, exercise, energy and motivation”, “but the depression is not where it normally is I think due to the pain” [I]<br>“muscle…softer and more pliable…I think that’s overall why I’ve been feeling so light and happier” [J]<br>“well the pain was the main one, but the depression. I think I’d prefer to get rid of the depression ‘cause once you get rid of that and you’re sleeping then the pain might slowly ease, because the last 3 nights it has, it obviously has ‘cause I’ve slept…it was the getting to sleep that was making my depression bad, not having any sleep” [K]<br>“But at the same time it’s like you know I’m not fighting with my mind so when I need to go to sleep, I fall asleep” [L]<br>“my mood’s ain’t been too bad, probably because I’ve been getting a bit more sleep” [M]<br><br>**Visit 18**<br>“My shoulders and my neck umm that’s not so bad but I think a lot of that is stress-related as well so and I believe because my mood has lifted in that respect that stiffness isn’t there all the time.” “[fatigue, tiredness and lethargy] quite bad on an afternoon. Um but that’s because maybe I’m just you know I haven’t got, I haven’t got to be anywhere so I think it’s a state of mind but as of Monday when I start a new job I think things will change again there because my mind’s more active” [A]<br>“anxiety been a little lower…but I don’t know again if that’s because I’m in less pain so I’m less like tensed up” [I]<br>“It’s been years and years. I mean it’s mainly down to pain but a lot of it is mainly down to you know state of mind as well…I mean the sex life is something what was non-existent like I say before, and that is one aspect what’s changed”, “yeah, and I think that sort of bridges across to the change in sex life as well, you know my motivation in trying new things…it’s just doing stuff what I wouldn’t normally try” [M]<br>“the wife’s picked up as well with my moods, I think ‘cause I ain’t been aching so much over the last few weeks. Generally, it’s like anything, if you feel rough, if you feel poorly you know, you just want to be left alone, where I just felt better in myself you know and I just hope this continues” [N]	

	Ability to cope with/push aside and ignore other symptoms	**Negative cases:**<br><br>**Visit 9**<br><br>**Visit 18**<br>"But sometimes if I push through it then the following day I pay for it" [C]<br><br>**Quotes:**<br><br>**Visit 9**<br>"I just feel very positive, umm, and I push to one side the tiredness" "tomorrow regardless of how I feel I have got things to do. I've gotta go and do my Mom's shopping so it's in, it's in your mind isn't it so. Pain you push to one side, tiredness you push to one side" "I sort of push myself to try and do something ... so you know the tiredness don't get to me too much.. I think if, if y'am your mind's occupied and you're doing things... You just gotta get on with it" [A]<br>"I have learnt to do everything in pain but after a little while so if the bad days are like continuous it really affects my mood, my energy levels – because it's constant and there's no break. Whereas only having one or two to me that's a big thing because I can kind of manage having 1 or 2 – and feel less…hated on my own body" [D]<br>"even with the pain that I've still got I can manage the pain that I've got" [G]<br>"I was working from home, but I found work harder going into the office….and I'm actually getting through the day without having to phone my boss and say 'look I need to go home and work from home, 'cause when I'm at home it's more comfortable, I walk around a lot more which helps with the pain, but no I've got through fine without saying I need to go home'" [K]<br><br>**Visit 18**<br>"having this condition is something that you've just gotta get on and live with" [A]<br>"it makes the pain um *(sighs)* less significant in a way because um all that other cloud around it is is lifting so you know I can I can manage it better now and I've got the energy to do that as well" [B]<br>"I think sometimes it's that knowing what I've got to do and evaluating how tired I actually am to how much work I've gotta do and whether I can push through it." "I can get on with what I want to. So if I have bad days it's kind of well actually, they're not gonna last forever. Whereas before it was like 'oh God, is it coming, how long's this gonna last for'. An' it's like the aches and pains generally have subsided really, really well. I'm still getting pain with me back an' there's nothing I can do about that" [C]<br>"'cause it's not a forward thought anymore it's just 'OK you are in pain *name* but you can still keep doing things'." [D]	A, B, C, D, F, G, I, K, M, N

		"I thought no I can stand this pain for a couple of hours longer" "because it's been that severe [previously] that it was unbearable I thought I can stand this a little bit longer" "I can't spend the rest of my life lying in this bed, thinking and lying there in pain. But you're gonna be in pain whether I'm sitting in the garden or lying down and that's what I thought" [F]<br>"I'm not dwelling on the pain so like it's made a difference that way" [I]<br>"I've noticed a couple of days of the week I've been able to walk more, I've been able to move more but then I'm relapsing…pain and fatigue….I've been tracking it on this, um steps, this is for the week, I mean the 20th July which was Wednesday I managed to do 9351 steps but yesterday I only managed 1900…there's a lot of dips but I mean there's a lot of peaks as well…the past week it's been better", "getting about the house, again, it's painful but I'm still managing to do it", "It's not going to cause me any more pain in doing it. I mean everything what everybody said, you know go swimming twice a week it doesn't really cause me extra pain it cause me fatigue more than anything, it just completely wears me out but like I say trying you know the you know the focus mits for my wife at kick boxing, taking the kids to their martial arts classes um you know um and things I probably wouldn't have done before" [M]<br>"[bend down] just little things like that yeah, don't get me wrong it's still hurts, but it's eased" [N]	
